# Novel Vitreous Substitutes in Animal and Human Models: A Systematic Review

**DOI:** 10.1155/bmri/8731510

**Published:** 2026-06-23

**Authors:** Danielle Solish, Lia Huo, Tony Shicheng Jin, Brandon Hall, Eunice L. You, Marko M. Popovic, Adam Forman, Molly Shoichet, Robert Devenyi, Brian G. Ballios, Peng Yan

**Affiliations:** ^1^ Department of Ophthalmology and Vision Sciences, University of Toronto, Toronto, Ontario, Canada, utoronto.ca; ^2^ Temerty Faculty of Medicine, University of Toronto, Toronto, Ontario, Canada, utoronto.ca; ^3^ Department of Ophthalmology and Visual Sciences, McGill University, Montreal, Quebec, Canada, mcgill.ca; ^4^ Department of Chemical Engineering and Applied Chemistry, University of Toronto, Toronto, Ontario, Canada, utoronto.ca; ^5^ Institute of Biomedical Engineering, University of Toronto, Toronto, Ontario, Canada, utoronto.ca

**Keywords:** device, hydrogel, liquid, polymers, systematic review, vitreous substitute

## Abstract

**Introduction:**

Vitreous substitutes are crucial adjuncts during vitreoretinal surgery for retinal detachment, macular holes, and vitreous hemorrhage. However, current vitreous substitutes pose limitations, including but not limited to their postoperative decrease in visual sharpness, nonfavorable refractive indices, and complications such as glaucoma, cataract, and retinal toxicity.

**Methods:**

We conducted a systematic review to evaluate the biological, physical, and clinical properties of novel experimental vitreous substitutes in animal and human models. We performed a systematic search on Ovid Medline, Embase, and Scopus from January 1, 1990, to October 10, 2025. Studies were included if they reported on any of the following three aspects: optical properties, biological interactions, or clinical outcomes.

**Results:**

Out of 11,045 search results, 37 studies investigating novel hydrogels (*n* = 15), polymers (*n* = 5), liquids (*n* = 5), and devices (*n* = 12) were included. Among these, 16/37 substitutes had > 80% success in tamponade of retinal detachments, 12/37 showed high tissue biocompatibility, 15/37 demonstrated low ocular inflammation, 13/37 exhibited optical properties similar to native vitreous, and 14/37 were associated with no significant changes in intraocular pressure.

**Conclusions:**

Novel experimental vitreous substitutes, such as UV‐CHA, Vitargus ABV‐1701, PanaceaGel SPG‐178, E10KDC18, or FCVB, show the most promising biological, optical, and physical properties for additional investigation as vitreous substitutes.

## 1. Introduction

Vitreous substitutes are crucial adjuncts during vitreoretinal surgery to treat conditions such as retinal detachment (RD), macular holes, and complications of diabetic retinopathy. In cases of RD, a key determinant of a positive outcome involves the type of vitreous substitute used as an endotamponade to attach the neurosensory retina back to the underlying retinal pigment epithelium (RPE) [1, 2].

Currently, common vitreous substitutes include sterile room air, expansile gasses such as sulfur hexafluoride (SF_6_), perfluoropropane (C_3_F_8_), and perfluoroethane (C_2_F_6_), liquids such as balanced salt solution (BSS), perfluorocarbon liquids (PFCLs), semifluorinated alkanes (SFAs), silicone oils, and heavy silicone oils [1]. These substitutes have favorable optical properties, are chemically inert, and serve as an effective tamponade. However, these current vitreous substitutes pose limitations in terms of postoperative decrease in visual sharpness, nonfavorable refractive indices, significant need for patient positioning, as well as complications including corneal toxicity, cataract, glaucoma, intraocular inflammatory reactions, emulsification, retinal toxicity, intraretinal layer disruption, and removal surgeries in the case of nonresorbable fluids [3, 4].

Given the inherent limitations with current vitreous substitutes, a search for a novel substitute is being undertaken. An ideal substitute would share similar biophysical properties as the native vitreous, including sharing the same filling function inside the vitreous cavity, similar viscoelastic properties, allowing the diffusion of metabolites, gasses, and drugs, being nonimmunogenic and inert to surrounding ocular structures, as well as maintaining normal intraocular pressure (IOP) [3, 4]. Perhaps most importantly, the postoperative substitute should have sufficient surface tension and density to act as a retinal tamponade. If biodegradation or bioresorption does occur, the substitute should break down into nontoxic by‐products that are easily absorbed by ocular tissues and be removed at a rate that allows for sufficient time for a robust internal tamponade effect. A key determinant of success is using a nonswelling material thereby preventing increased IOP, which can lead to glaucoma. In addition, an optically transparent substitute with a refractive index similar to that of the native vitreous would facilitate vision, and an injectable substitute would provide ease of delivery during standard vitreoretinal surgery [2–4].

Over the past three decades, research in animal and human models has focused on finding the ideal vitreous substitute. To date, there has been no comprehensive systematic review of the research progress on experimental vitreous substitutes. The aim of this systematic review is to examine the optical, biological, and clinical outcomes of experimental vitreous substitutes evaluated in animal and human models over the last 30 years.

## 2. Methods

### 2.1. Eligibility Criteria

A systematic search of the literature was conducted on Ovid Medline, Embase, and Scopus between January 1st 1990 and October 10th 2025 to identify relevant experimental substitute studies. A search strategy, developed in conjunction with an academic librarian, was performed independently by two authors (T.S.J. and E.L.Y.) to identify relevant studies. This review′s protocol was registered on PROSPERO (CRD42022354865) prior to the commencement of the study. The search strategy results were combined, and duplicates were removed. The selection of studies was outlined according to Preferred Reporting Items for Systematic Reviews and Meta‐Analyses (PRISMA) guidelines.

Primary research studies were included if:

1. A vitreous substitute was considered “novel.” This is defined as being studied as part of experimental research and also not being an established vitreous substitute such as room air, expansile gasses such as SF_6_ and C_3_F_8_, liquids such as BSS, PFCLs, SFAs, silicone oils, and heavy silicone oils unless being compared with an experimental substitute.
2. The novel vitreous substitute was evaluated in vivo in an animal or human model with at least *n* ≥ 10 eyes.
3. The vitreous substitute reported on at least one of the following:
  a. Optical properties of the vitreous substitute, such as refractive index.
  b. Biological interactions, including physiological effects such as ocular tissue toxicity.
  c. Clinically relevant outcomes, such as efficacy of retinal tamponade, electroretinogram (ERG) measurements, and surgical complications, of the novel vitreous substitute.

Studies were excluded if they were nonprimary research articles, such as reviews and practice guidelines, nonpublished and/or nonpeer‐reviewed resources, or non‐English articles. Beyond the database search, the reference lists of all included studies were reviewed to identify further relevant studies.

### 2.2. Study Selection and Data Extraction

Two reviewers (T.S.J. and E.L.Y.) independently screened titles and abstracts for eligibility criteria in duplicate. Full‐text manuscripts were reviewed to determine final inclusion. Any discrepancies were resolved by consensus decision of the same two authors (T.S.J. and E.L.Y.). A predetermined form was filled out with variables of interest, which included date of publication, journal, type of subjects, number of subjects, type of vitreous substitute, optical properties, biological interactions, and clinical outcomes, if reported.

### 2.3. Risk of Bias Evaluation

The Systematic Review Centre for Laboratory Animal Experimentation (SYRCLE) Risk of Bias Assessment tool [5] and the Cochrane Collaboration Risk Of Bias In Non‐randomized Studies of Interventions I (ROBINS‐I) tool [6] for human studies were used to evaluate the risk of bias of all participants.

### 2.4. Outcome Evaluation

Results were reported using descriptive statistics. The main outcomes are (1) biocompatibility as measured by cell toxicity, morphological integrity, and so on., (2) physical properties such as optical properties, intraocular tamponade efficacy, intraoperative surgical parameters, and (3) clinical outcomes including postoperative visual acuity, retinal reattachment rate, IOP, other postoperative complications such as cataract, glaucoma, uveitis, retinal toxicity, and so on, and patient‐reported outcomes, as available.

### 2.5. Data Availability

The authors confirm that the data supporting the findings of this study are available within the article and tables.

### 2.6. Table of Contents

Current vitreous substitutes pose limitations in terms of optical outcomes post vitreoretinal surgery. This systematic review examines properties of novel experimental vitreous substitutes. It concludes that UV‐CHA, Vitargus ABV‐1701, PanaceaGel SPG‐178, E10KDC18, or foldable capsular vitreous body (FCVB) pose the most promise for their biological, optical, and physical properties.

## 3. Results

### 3.1. Study Selection

**Figure 1:**
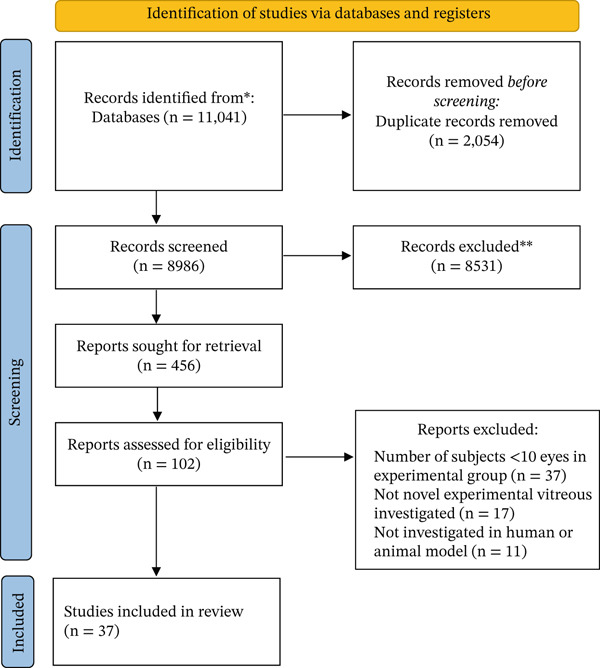
PRISMA flow diagram of study identification and selection.

The literature search identified 11,045 potentially eligible studies. After exclusion of duplicates and review of abstracts, 10,591 studies were excluded (Figure 1). On full‐text review, further studies were excluded, resulting in 37 final included studies. The primary reason for exclusion was studies that did not evaluate novel vitreous substitutes or studies with < 10 eyes evaluated with a novel vitreous substitute.

### 3.2. Study Characteristics

Included studies are summarized in Table 1. A total of 37 studies published between 1990 and 2025 met all inclusion criteria. Experimental vitreous substitutes investigated across these studies comprised polymers (*n* = 19), liquids (*n* = 6), and devices (*n* = 12). Studies published within the last 3 years that did not meet the minimum sample size criterion (*n* < 10 eyes) are summarized separately in Table 2 to provide additional context on emerging work in the field.

**Table 1:** Summary of included studies investigating experimental vitreous substitutes from 1990–2025.

**Study**	**Authors**	**Aim**	**Study Subjects (e.g. Human, Monkey, Rabbits, etc)**	**Total Eyes # (n=)**	**Eyes that received Experimental Subject # (n=)**	**Type (Device, Hydrogel, Polymer, Smart Gel, etc)**	**Substitute Description**	**Comparator Substitute (if any)**	**Disease studied (retinal detachment, endophthalmitis, proliferative vitreoretinopathy, etc)**	

Stable oxime‐crosslinked hyaluronan‐based hydrogel as a biomimetic vitreous substitute	Baker A., and Cui, H. and Ballios, B. and Ing, S. and Yan, P. and Wolfer, J. and Wright, T. and Dang, M. and Gan, N. and Cooke, M. and Ortin‐Martinez, A. and Wallace, V. and van der Kooy, D. and Devenyi, R. and Shoichet, M.	To evaluate the use of cross‐linked HA‐oxime as a choice for a vitreous substitute for the treatment of retinal detachment.	Rabbits	n=40	n=40	Hydrogel	A new oxime hydrogel vitreous substitute was engineered by chemically modifying Hyaluron, which is naturally abundant in the vitreous of the eye to crosslink with poly(ethylene glycol)‐tetraoxyamine via oxime chemistry.	Silicone oil	Retinal detachment	
Vitreous Substitutes Following Vitrectomy Surgery	Chang, A.	Phase I clinical trial to evaluate the safety and tolerance of Intravitreal Vitargus.	Human	n=11	n=11	Hydrogel	Hydrogel made of oxidized HA crosslinked with adipic acid dihydrazide (ADH).	None	Retinal detachment or vitreous hemorrhage	
Design of an injectable in situ gelation biomaterials for vitreous substitute	Annaka, M. and Mortensen, K. and Vigild, M. E. and Matsuura, T. and Tsuji, S. and Ueda, T. and Tsujinaka, H.	To design an injectable in situ gelation biomaterial for vitreous substitution	White rabbits	n=10	n=10	Polymer (forms a transparent gel in the vitreous cavity)	Poly(ethylene glycol) end‐capped with an octadecyl group: E10KDC18 ‐ E10KDC18 were obtained via Williamson reaction of an octadecyl bromide on metalated PEG. Thermosensitive amphiphilic polymer.	None	Retinal detachment	
Safety of medium‐chain triglycerides used as an intraocular tamponading agent in an experimental vitrectomy model rabbit	Auriol, S. and Mahieu, L. and Brousset, P. and Malecaze, F. and Mathis, V.	To evaluate safety of medium‐chain triglycerides used as a possible intraocular tamponading agent.	Rabbits	n=28	n=28	medium‐chain triglyceride	Medium‐chain triglycerides. The solution of MCT used in this experiment was purchased from Sasol GmbH (Germany) under brand name Miglyol 812. Medium‐chain triglycerides were repackaged and sterilized by Novagali Pharma. The filled vials were sterilized by steam during 20 minutes at 120°C.	N/A	Posterior vitreous detachment	
A cross‐linked hyaluronic acid hydrogel (Healaflow) as a novel vitreous substitute	Barth, H. and Crafoord, S. and Andréasson, S. and Ghosh, F.	In this study, a hydrogel of cross‐linked sodium hyaluronic acid (Healaflow®) is evaluated for use as a novel vitreous substitute.	Pigmented rabbits	n=12	n=12	Hydrogel	‐Healaflow is a commercially available transparent hydrogel, clinically used in glaucoma filtering surgery as a space‐filler, and to limit postoperative fibrosis. ‐It consists of over 97% water, sodium hyaluronic acid (22.5 mg/ml) of non‐animal origin cross‐linked with BDDE (1.4‐Butanediol diglycidyl ether), and phosphate‐ and NaCl‐salts to maintain physiological pH (7.0) and osmolarity (305 mOsm/kg).	N/A	N/A	
Perfluoro‐n‐octane as a temporary intraocular tamponade in a staged approach to manage complex retinal detachments	Barthelmes, D. and Chandra, J.	To evaluate outcomes in patients with complex retinal detachments (RD) with proliferative vitreoretinopathy (PVR) requiring retinectomy using a staged approach utilizing perfluoro‐n‐octane (PFO) as a short‐term postoperative intraocular tamponade.	Human	n=17	n=17	Polymer	PFO ‐ Perfluoro‐n‐octane	Silicone oil	Complicated retinal detachment	
New biodegradable networks of poly(N‐vinylpyrrolidinone) designed for controlled nonburst degradation in the vitreous body	Bruining, M. J. and Edelbroek‐Hoogendoorn, P. S. and Blaauwgeers, H. G. and Mooy, C. M. and Hendrikse, F. H. and Koole, L. H.	To report on the design and synthesis of a new family of biodegradable biomaterials, which may be useful to overcome some of the typical problems of poly(lactic acid) or poly(D,L‐lactic‐co‐glycolic acid) in intraocular applications.	Pigmented rabbits	n=19	n=19 (A=5, B=5, C=5, D=1, E=1, F=1, G=1)	Hydrogel	‐All polymeric networks studied in this work are copolymers of Polymers of N‐vinylpyrrolidinone (NVP)‐ a series of 3‐dimensional networks of poly (NVP) and crosslinker 1. ‐Studied compositions included A (1:10.5 / BC:NVP; 5 eyes), B (1:105 / BC:NVP; 5 eyes), C (1:1000 / BC:NVP; 5 eyes), D (Poly[NVP]; 1 eye), E (1:10.5 / SC:NVP; 1 eye), F (1:105 / SC:NVP; 1 eye), and G (1:1000 / SC:NVP; 1 eye).	Materials E‐G: NVP crosslinked with a nondegradable monomer, tetraethylene glycol dimethacrylate (TEGDMA).	N/A	
Functional evaluation of a novel vitreous substitute using polyethylene glycol sols injected into a foldable capsular vitreous body	Chen, H. and Feng, S. and Liu, Y. and Huang, Z. and Sun, X. and Zhou, L. and Lu, X. and Gao, Q.	To evaluate whether PEG injected into FCVB can prolong the duration time of the PEG and serve as a long‐term vitreous substitute after a long period (180 days) of implantation	New Zealand Albino Rabbits	n=30	n=9 (with FCVB)	Device‐ FCVB	Polyethylene Glycol injected into a foldable capsular vitreous body (FCVB). PEG was prepared in sterile PBS with molecular weights of 400 kDA. 2.5, 5 and 7.5% solution of PEG sols were evaluated.	Balanced salt solution (BSS)	Retinal detachment	
Clinical device‐related article evaluation of morphology and functions of a foldable capsular vitreous body in the rabbit eye	Chen, J. and Gao, Q. and Liu, Y. and Ge, J. and Cao, X. and Luo, Y. and Huang, D. and Zhou, G. and Lin, S. and Lin, J. and To, C. H. and Siu, A. W.	The FCVB was designed to mimic natural vitreous morphology, to further evaluate its physiological functions compared with traditional silicone‐oil substitutes, in an established animal model of PVR.	New Zealand Albino Rabbits	n=40	n=20	Device‐ FCVB	Foldable Capsular Vitreous Body (FCVB): mirror steel mold that consisted primarily of an upper composite die, a lower composite die, and the core. BSS was injected into the capsule through the tube device and the pressure was adjusted by BSS injection.	Silicone oil substitution	Retinal detachment	
Reattachment after foldable capsular vitreous body implantation in severe retinal detachment eyes	Chen, S. and Tian, M. and Zhang, L. and Hu, C. and Liu, K. and Qin, B. and Liu, S.	To evaluate the clinical effectiveness and safety of foldable capsular vitreous body (FCVB) implantation for severe retinal detachment.	Human	n=27	n=27	Device‐ FCVB	The FCVB is made from a Food and Drug Administration‐registered, nontoxic, medical grade silicone rubber with good biocompatibility and stability. The capsule shape is determined by computer simulation of the human and rabbit vitreous cavities.	N/A	Severe retinal detachment: severe ocular trauma (n=26), recurrent retinal detachment (n=1)	
A novel vitreous substitute of using a foldable capsular vitreous body injected with polyvinylalcohol hydrogel	Feng, S. and Chen, H. and Liu, Y. and Huang, Z. and Sun, X. and Zhou, L. and Lu, X. and Gao, Q.	To evaluate the long‐term retina support and biocompatibility of cross‐linked PVA hydrogel combined with FCVB via implanting it into the vitreous cavity of rabbit.	New Zealand Albino Rabbits	n=18	n=12 (6 PVA, 6 PVA+FCVB, 6 BSS)	Device (FCVB) with Hydrogel	Poly‐vinyl alcohol (PVA). 3% of PVA solution prepared in ultra‐purified water and were stirred until fully dissolved at 80 °C. Then, the solution was irradiated by c‐rays (7 kGy, Co60) to form a hydrogel.	FCVBs with the 3% hydrogel were compared to a 3% PVA hydrogel alone or a balanced salt solution (BSS)	N/A	
Evaluation of a viscoelastic solution of hydroxypropyl methylcellulose as a potential vitreous substitute	Fernandez‐Vigo, J. and Refojo, M. F. and Verstraeten, T.	The object of this research was to determine HPMC dynamics in the rabbit vitreous cavity, and its effect on the morphology of cultured human retinal pigment epithelial cells.	Rabbits (mixed‐breed, pigmented rabbits)	n=32	n=16	Polymer	Hydroxypropyl Methylcellulose (HPMC): an inert polymer that forms a viscoelastic solution in aqueous material. A 2.2% solution of HPMC (MW: 86,000 daltons) of 6,000 centistokes viscosity	None	Complicated retinal detachments	
A new strategy to replace the natural vitreous by a novel capsular artificial vitreous body with pressure‐control valve	Gao, Q. and Mou, S. and Ge, J. and To, C. H. and Hui, Y. and Liu, A. and Wang, Z. and Long, C. and Tan, J.	To replace the natural vitreous by a novel capsular artificial vitreous body with a pressure‐control valve.	Rabbits (New Zealand albino rabbits)	n=40		Device	The basic material belongs to tailor‐made modified silicone rubber elastomer. It is a transparent macromolecule cross‐linking polymer of polyvinylsiloxane and polyhydrosiloxane with good mechanical properties and biocompatibility. PBS is injected into the capsule and inflate to support the retina and control IOP through the tube‐valve system.	None	No specific disease ‐ just in general for PPV surgery (including but not limited to diabetic retinopathy, age related macular degeneration, retinal detachment and traumatic retinopathy)	
Effect on rabbits′ intraocular structure by cross‐linked hyaluronic formations as vitreous substitute	Gong, Yan and Chen, Kan and Wu, Yue and Guo, Xiao‐Hong and Zhang, Tao	To develop a new material for retina filling and to investigate its effect on intraocular structure and histocompatibility in rabbit eyes	Rabbits (New Zealand white rabbits)	n=40	n=20 (3 groups: 24mg/mL HA, 40mg/mL HA, Normal saline) ; unclear how many were in each group.	Hydrogel	Cross‐linked HA was produced with two specifications of 24 mg/mL and 40 mg/mL.	0.9% saline (mean normal control)	N/A	
Perfluorohexylethan (O62) as ocular endotamponade in complex vitreoretinal surgery	Hoerauf, H. and Roider, J. and Kobuch, K. and Laqua, H.	To investigate the safety and performance of perfluorohexylethan (O62), a partially fluorinated alkane, as an intraoperative tool and heavy ocular endotamponade in complex vitreoretinal surgery.	Human	n=11	n=11	Fluoropolymer	O62‐ The partially fluorinated alkane used was O62. The chemical formula of this compound is C6F13C2H5,. For O62, 6 and 2 indicate the number of fluorinated and hydrogenated carbon atoms, respectively, of the molecule. O62 has a purity of 100%.	N/A	Proliferative vitreoretinopathy, rhegmatogenous retinal detachment with inferior tears, and inferior giant tears	
Long‐Term Biocompatibility of a Highly Viscously Thiol‐Modified Cross‐Linked Hyaluronate as a Novel Vitreous Body Substitute	Hurst, J. and Rickmann, A. and Heider, N. and Hohenadl, C. and Reither, C. and Schatz, A. and Schnichels, S. and Januschowski, K. and Spitzer, M. S.	To evaluate the long‐term biostability and histocompatibility of a highly viscously thiol‐modified crosslinked hyaluronate (TCHA) in a rabbit model after vitrectomy.	Rabbits (Chinchilla bastard rabbits)	n=32	n=18	Hydrogel (prepared as an injectable gel implant)	Thiol‐Modified Crosslinked Hyaluronate (TCHA): clear, viscous hydrogel, free of visible particles, homogenized and prepared as injectable gel implant, which had been steam sterilized. Formulations were prepared in physiological phosphate buffer and contained 2.2% HA.	N/A	No specific disease ‐ treatment of complicated retinal and vitreous diseases in general	
The feasibility study of an in situ marine polysaccharide‐based hydrogel as the vitreous substitute	Jiang, X. and Peng, Y. and Yang, C. and Liu, W. and Han, B.	In the present study, an injectable in situ‐forming hydrogel was prepared through Schiff base reaction between HPCTS and ADA. The physical properties, rheological properties, and cytotoxicity of the hydrogel were studied. Subsequently, the hydrogel was injected into the vitreous cavity of rabbit eyes following vitrectomy to examine its feasibility and biosafety as a potential vitreous substitute.	Rabbits (New Zealand white rabbits)	n=20	n=10	Hydrogel	An injectable in situ‐forming hydrogel was prepared through Schiff base reaction between Hydroxypropyl chitosan (HPCTS) and Alginate Dialdehyde (ADA). The HPCTS‐ADA hydrogel was obtained by mixing equal volume of the two filtered solutions at 37°C.	N/A	N/A	
Application of thermo‐setting gel as artificial vitreous	Katagiri, Y. and Iwasaki, T. and Ishikawa, T. and Yamakawa, N. and Suzuki, H. and Usui, M.	To investigate the safety of intravitreous injection of a thermo‐setting gel (TG) to determine whether TG can be used as artificial vitreous.	Rabbits (Male Japanese white rabbits)	n=20	n=10	Thermo‐Setting Gel	The main ingredient of thermo‐setting gel is methylcellulose which has the property of reversible sol‐gel transformation at 55 °C. Various thermo‐setting gels were prepared by changing the ratio of the ingredients,and the optimal preparation, WTG‐127 (Wakamoto Pharmaceutical, Tokyo, Japan) was used. WTG‐127 gelates at the relatively low temperature of 36°C, which is almost equivalent to body temperature, and also retains transparency even upon gelation	Blue WTG	N/A	
Foldable Capsular Vitreous Body Implantation for Complicated Retinal Detachment Caused by Severe Ocular Trauma	Li, M. and Tang, Y. and Li, S. and Zhang, Z. and Guan, L. and Li, J. and Xu, J. and Ji, S.	To implant FCVB into the vitreous cavity after PPV to study the effectiveness, safety, and psychological impact in the treatment of complicated retinal detachment caused by severe ocular trauma.	Human	n=28	n=28	Device ‐ FCVB	‐ Transparent macromolecule cross‐linking polymer of polyvinylsiloxane and poly‐ hydrosiloxane with good mechanical properties and biocompatibility and has been proven to be nontoxic to mouse fibroblast cells. ‐ Optical properties transmittances are 92%, hazes are 5.74%, and spectral transmittance is 97%.	None	Complicated retinal detachment caused by severe ocular trauma.	
Evaluation of the flexibility, efficacy, and safety of a foldable capsular vitreous body in the treatment of severe retinal detachment	Lin, X. and Ge, J. and Gao, Q. and Wang, Z. and Long, C. and He, L. and Liu, Y. and Jiang, Z.	To determine the flexibility, efficacy, and safety of a novel foldable capsular vitreous body (FCVB) in the treatment of severe retinal detachment in human eyes.	Human	n=11		Device ‐ FCVB	‐ The FCVB consists of a thin vitreous‐shaped capsule with a tube‐valve system made with computer and industrial technology. After the folded body is installed in the eye, balanced salt solution is then injected into the capsule, inflating it to support the retina. The tube–valve system allows control of IOP. ‐ It is composed of liquid silicone rubber, which is a non‐toxic and stable material.	None	RD	
Retinal‐detachment repair and vitreous‐like‐body reformation via a thermogelling polymer endotamponade	Liu, Z. and Liow, S. S. and Lai, S. L. and Alli‐Shaik, A. and Holder, G. E. and Parikh, B. H. and Krishnakumar, S. and Li, Z. and Tan, M. J. and Gunaratne, J. and Barathi, V. A. and Hunziker, W. and Lakshminarayanan, R. and Tan, C. W. T. and Chee, C. K. and Zhao, P. and Lingam, G. and Loh, X. J. and Su, X.	To develop a thermogelling agent that fulfils the clinical requirements of a tamponade agent and resemble the consistency of human vitreous humour.	Rabbits	n=41	n=25	Hydrogel	EPC‐7% thermogel has 2 unique functions: ‐ First, it acts as endotamponade agent for immediate post‐op period by generating sufficient surface tension to bridge across retinal break and re‐appose detached retina through surface tension enabling the breaks to be sealed. ‐ Second, it acts as a biodegradable scaffold implant for in vivo restoration of a vitreous‐like body, thus obviating the need for removal surgery.	EPC‐ 3% n = 6, EPC‐7% n = 15, EPC‐12% n = 4, Pluronics F127 20% n = 4, operated control (BSS) n = 12	N/A	
Evaluation of collagen gel and hyaluronic acid as vitreous substitutes	Nakagawa, M. and Tanaka, M. and Miyata, T.	To evaluate alkaline‐solubilized collagen, hyaluronic acid (HA), and a substance formed from mixing both materials as vitreous substitutes in the rabbit.	Rabbits	n=28	n=24	Polymer	Alkaline‐solubilized collagen, high‐molecular‐weight NaHA (obtained through a fermentation process), and a gel formed by mixing both materials.	8 rabbit eyes each were used to study FITC‐collagen. FL‐HA, and the mixture. 4 eyes served as controls.	RD	
Super‐fast in situ formation of hydrogels based on multi‐arm functional polyethylene glycols as endotamponade substitutes	Ran, R. and Shi, W. and Gao, Y. and Wang, T. and Ren, X. and Chen, Y. and Wu, X. and Cao, J. and Zhang, M.	To fabricate a fast in situ forming PEG‐engineered hydrogel by an easy reaction process without cytotoxicity for achieving the excellent vitreous filling.	Rabbits	n=46	n=34	Hydrogel	‐Excellent biocompatibility, hydrophilicity, stability and anti‐protein adsorption. The modification of hydroxyl end groups in PEG into several other functional groups makes it possible for fabricating in situ forming hydrogels.	A total of 46 chinchilla rabbits were randomized into 5 groups, operated control group (BSS, n = 12), AVB groups (c = 9 mg mL−1, n = 16; c = 10 mg mL−1, n = 8; c = 20 mg mL−1, n = 8), and RD group (c = 9 mg mL−1, n = 2).	N/A	
Experimental vitreous replacement with perfluorophenanthrene	Ratiglia, R. and Berti, E. and Galimberti, D. and Bindella, A. and Schweizer, F. and Marchi, L. and Rossi, A.	To check whether PFP is tolerated as a tamponade in the eye or damages the retina, and if any such damage is due to toxicity or to emulsification which may stimulate phagocytosis.	Rabbits	n=24 rabbits, 36 eyes (12 controls)	n=24	Polymer	The perfluorocarbons commonly used in vitreoretinal surgery are perfluorodecaline and perfluorophenanthrene (PFP). PFP has less retinal toxicity than perfluorodecaline, though long‐term permanence in the eye induces considerable alterations of the retinal architecture.	Control	Unknown	
Bioinspired Fibrillary Hydrogel with Controlled Swelling Behavior: Applicability as an Artificial Vitreous	Santhanam, S. and Shui, Y. B. and Struckhoff, J. and Karakocak, B. B. and Hamilton, P. D. and Harocopos, G. J. and Ravi, N.	‐To study the osmotic swelling behavior of the original 11 formulations of the two‐component hydrogels. ‐To evaluate the applicability of the biomaterial as an artificial vitreous in a 1 month study in Dutch‐belted rabbits (in the 2 formulations picked from the abovementioned 11).	Rabbits	n=32 rabbits / eyes	n=22	Hydrogel	An injectable two‐component hydrogel composed of a fibrillary gellan and a semiflexible polyelectrolyte, poly[methacrylamide‐co‐(methacrylic acid)], both endowed with thiol cross‐linkers.	N/A	N/A	
Efficacy of two different thiol‐modified crosslinked hyaluronate formulations as vitreous replacement compared to silicone oil in a model of retinal detachment	Schnichels, S. and Schneider, N. and Hohenadl, C. and Hurst, J. and Schatz, A. and Januschowski, K. and Spitzer, M. S.	To evaluate the efficacy of a vitreous substitute based on tVBS in a model of RD in comparison to current standard treatment with silicone oil. Prior to their surgical use, the generated hydrogels were characterized with respect to optical and rheological properties and accordingly two tVBS hydrogels with differing viscosities were chosen for further evaluation.	Pigmented rabbits	n=24 experimental (rabbits); 48 eyes total (experimental + control)	n=16	Hydrogel	In the present study a vitreous substitute based on cross‐linked thiolated HA (tVBS) was evaluated. The thiol‐modified polymer is able to build stable hydrogels by natural formation of disulfide bridges and thus does not require addition of chemical cross‐linkers or other manipulation.	Control (contralateral eye) and silicone oil	RD	
The cross‐linked biopolymer hyaluronic acid as an artificial vitreous substitute	Schramm, C. and Spitzer, M. S. and Henke‐Fahle, S. and Steinmetz, G. and Januschowski, K. and Heiduschka, P. and Geis‐Gerstorfer, J. and Biedermann, T. and Bartz‐Schmidt, K. U. and Szurman, P.	To compare two ways to create a three‐dimensional hydrogel with long‐term biostability for a vitreous substitute based on cross‐linked hyaluronic acid (CHA).	Rabbits	n=12 rabbits / 24 eyes (i.e., 12 control eyes)	n=12	Hydrogel	HA is a part of the human vitreous, and so it is predestined to be a basic material for the development of a 3‐d vitreous substitute. 12,13 HA is a glycosaminoglycan co‐polymer of d‐glucuronic acid and N‐acetyl‐d‐glucosamine, which are connected through alternating *β*‐1,4 and *β*‐1,3 glycosidic bonds. The axial hydrogen atoms form a nonpolar, hydrophilic face, thereby creating a twisting ribbon structure.	Control (contralateral eye)	RD	
Outcomes of surgery for retinal detachment associated with proliferative vitreoretinopathy using perfluoro‐n‐octane: A multicenter study	Scott, I. U. and Flynn Jr, H. W. and Murray, T. G. and Feuer, W. J.	The current study represents the largest reported series of patients with RD associated with proliferative vitreoretinopathy managed with PPV and intraoperative perfluoro‐ carbon liquid and was designed to report VA and anatomical outcomes, as well as complications, and to investigate clinical features associated with outcomes.	Human	n=555	n=555	Polymer	Because of their optical clarity, high specific gravity (1.76 for perfluoro‐n‐octane [PFO] and 2.03 for perfluoroperhydrophenanthrene), low viscosity (0.69 cen‐ tistokes at 25 C for perfluoro‐n‐octane and 8.03 centistokes for perfluoropherhydrophenanthrene), and immiscibility in water, perfluorocarbon liquids are often helpful during repair of complex retinal detachment to displace subretinal fluid and blood anteriorly (thereby often obviating the need for a posterior drainage retinotomy), unfold the retina in giant retinal tear cases, and to provide countertraction and retinal stabilization during membrane peeling in eyes with PVR.	None	RD with PVR	
Rabbit study of an in situ forming hydrogel vitreous substitute	Swindle‐Reilly, K. E. and Shah, M. and Hamilton, P. D. and Eskin, T. A. and Kaushal, S. and Ravi, N.	To report the preparation of a copolymeric hydrogel that can be injected via a small‐gauge needle in liquid form and undergo gelation within the vitreous cavity in the presence of physiologic oxygen concentration. Further, the mechanical and optical characteristics of the hydrogel were extensively evaluated before their use in vivo in an attempt to match their properties to that of the natural vitreous humor.	Black rabbits	n=14 rabbits / 28 eyes (14 controls)	n=10	Hydrogel	Thiol‐containing, in vivo forming hydrogel using copoly(acrylamide). 2 concentrations: 2% and 3%	The left eye served as the control in all animals and was harvested at the time the right eye was enucleated.4 right eyes were filled with air post PPV	N/A	
F6H8 as an intraoperative tool and F6H8/silicone oil as a postoperative tamponade in inferior retinal detachment with inferior PVR	Tosi, G. M. and Marigliani, D. and Bacci, T. and Romeo, N. and Balestrazzi, A. and Martone, G. and Caporossi, T.	To analyze the outcomes of patients with inferior RD complicated by inferior PVR, who were operated on using F6H8 as an intraoperative tool to flatten the retina and in whom a direct partial exchange between F6H8 and SO 1000 cSt was performed. The eyes were tamponed with different quantitative ratios of F6H8 and SO (70/30, 60/40, 50/50, 40/30, and 30/70).	Human	n=22	n=22	Polymer	Perfluorohexyloctane (F6H8) has a density of 1.3 g/cm^3. Although its tolerability and biocompatibility have been demonstrated in the experimental animal and in human eyes, early F6H8 dispersion and emulsification with consequent inflammatory responses have frequently been reported. The use of SO in combination with FALKs (heavy silicone oil, HSO), thereby increasing the viscosity of FALKs, has been suggested to reduce emulsification. 4 different prefabricated mixtures of FALKs and SO of varying specific gravities and viscosities are now available: Oxane HD, Densiron 68, HWS 46–3000, and HWS 45–3000. All these mixtures include high viscosity SO, ranging from 5000 to 100000cSt. While encouraging results have been published, difficulties associated with the intraoperative handling of the substance (e.g., air‐heavy tamponade exchange, heavy liquid‐heavy tamponade exchange, or heavy tamponade removal), as well as postoperative side effects, have also been reported.	None	Inferior retinal detachment with inferior PVR	
A self‐assembling peptide gel as a vitreous substitute: A rabbit study	Uesugi, K. and Sakaguchi, H. and Hayashida, Y. and Hayashi, R. and Baba, K. and Suganuma, Y. and Yokoi, H. and Tsujikawa, M. and Nishida, K.	To evaluate a self‐assembling peptide gel as a potential vitreous substitute	Rabbits	n=21 (15 Gel, 6 BSS)	n=15	Hydrogel	A self‐assembling peptide, SPG‐178, spontaneously assembles itself into nanofibers, creating a stable *β*‐sheet structure in water. The fibers then spontaneously build themselves into a 3‐d mesh net resulting in a clear hydrogel (PanaceaGel; Menicon Co., Ltd., Nagoya, Japan). When this self‐assembling peptide gel is broken through physical force, it temporarily enters a sol state, then reassembles itself back into a gel. The gel is highly transparent and can be sterilized with an autoclave.	6 rabbits underwent PPV and replacement with a balanced salt solution (BSS plus; Alcon), and served as controls.	N/A	
Antifouling Super Water Absorbent Supramolecular Polymer Hydrogel as an Artificial Vitreous Body	Wang, H. and Wu, Y. and Cui, C. and Yang, J. and Liu, W.	To evaluate a poly(N‐acryloyl glycinamide‐co‐carboxybetaine acrylamide) (PNAGA‐PCBAA) supramolecular copolymer (SP) hydrogel‐based vitreous substitute, which could be injected by shear‐thinning at room temperature through a 22G needle, and quickly gelled in vitreous cavity at body temperature without resorting heating or any chemical reaction. We will demonstrate that this SP hydrogel artificial vitreous body can maintain high transparency, stability, self‐adjustability, and ultralow fibrotic response in recovering its biological functions.	Rabbits	n=20 (4 groups: Normal, Control (sham‐operated group), PNAGA, and PNAGA‐PCBAA‐10‐4 (five rabbits per group).)	n=10	Hydrogel	In this work, in order to construct a highly stable and anti‐ fouling vitreous substitute, we prepared PNAGA‐PCBAA hydrogels through photoinitiated copolymerization. The molecular and network structure was depicted in Scheme 1. 1 The H NMR spectra presented the characteristic peaks of NAGA and CBAA monomer units, suggesting the formation of copolymer. We prepared a series of aqueous solutions of NAGA and CBAA, which were subjected to photoinitiation. We found that the gelation occurred when the initial concentration of monomers was above 3 wt%. One interesting phenomenon is that CBAA monomer powder could absorb moisture from air and turn into droplets within 30 min, assuming a hygroscopic characteristic due to the superhydrophilic nature of the CBAA monomer. This excellent water holding ability will be greatly beneficial for ophthalmic transplantation.	4 groups: Normal, Control (sham‐operated group), PNAGA, and PNAGA‐PCBAA‐10‐4 (5 rabbits per group).	N/A	
Biocompatibility and retinal support of a foldable capsular vitreous body injected with saline or silicone oil implanted in rabbit eyes	Wang, P. and Gao, Q. and Jiang, Z. and Lin, J. and Liu, Y. and Chen, J. and Zhou, L. and Li, H. and Yang, Q. and Wang, T.	To evaluate over a 180‐day period the biocompatibility and retinal support of a foldable capsular vitreous body injected with either saline or silicone oil implanted in rabbit eyes.	Rabbits	n=23 (18 FVCB, 5 BSS)	n=18	Device ‐ FCVB	FCVB	The FCVBs were implanted into the vitreous cavity of the rabbit eyes (n = 18). Silicone oil tamponade (Acri.Sil‐ol 5000; Acri.Tec, GmbH, Berlin, Germany) was used as the control group (n = 5). In the FCVB‐implanted eyes, either saline (n = 9) or silicone oil (n = 9) was injected into the FCVB capsule to support the retina.	PVR	
Outcomes of a Foldable Capsular Vitreous Body Implantation: A Retrospective Study	Xu, X. and Ge, H. and Li, J. and Shang, W. and Ji, Y. and Yang, W. and Li, K.	To investigate a method for implanting an FCVB, its postoperative efficacy, and clinical value.	Human	n=18		Device ‐ FCVB	FCVB was designed to change the traditional supporting mode of the retina with a 360° arc solid pressure effect and isolated the SO with the capsule of FCVB, which is a brand‐new therapy strategy. Since that, FCVB implantation has greatly reduced the incidence of complications. Furthermore, patients were did not need to maintain prone posture after surgery. Silicone oil can also be extracted through the drainage valve of the FCVB or injected with normal saline and silicone oil to regulate IOP.	None	Severe ocular rupture with retinal and choroid injury, visual acuity below 0.05	
Feasibility study of chitosan as intravitreous tamponade material	Yang, H. and Wang, R. and Gu, Q. and Zhang, X.	To investigate the possibility of chitosan as vitreous filling material, this study was designed to investigate retina, ciliary body, lens and cornea morphology changes, intraocular pressure and intraocular inflammatory factors fluctuating after chitosan intravitreous tamponade.	Chinchilla rabbits	n=30	n=12	Polymer	‐ Chitosan derives from the natural high molecular com‐ pound chitin extracted from the shell of crustaceans, an aminoglucose polymer from N‐acetylglucosamine polymer per de‐N‐acetyl. It is one of four generally accepted biomedical materials worldwide, which has good bio‐ compatibility, biodegradability, antibiosis, hemostasis and promotion of peri‐nerve regeneration. It can inhibit the proliferation of fibroblasts, and is an ideal drug delivery carrier. ‐ Clinically, chitosan has been widely used in surgical operations on articular cavity, abdominal cavity and pelvic cavity, and satisfactory effectiveness has been obtained in preventing postoperative adherence.	Sodium hyaluronate	Unknown	
Preliminary study on retinal vascular and oxygen‐related changes after long‐term silicone oil and foldable capsular vitreous body tamponade	Yang, W. and Yuan, Y. and Zong, Y. and Huang, Z. and Mai, S. and Li, Y. and Qian, X. and Liu, Y. and Gao, Q.	To investigate the morphological changes of retinal vessels and the relevant hypoxia cytokines’ expression vari‐ ation in silicone oil tamponade‐ and FCVBs combined with silicone oil that are implanted into the vitreous cavity of rabbits for 180 days.	Rabbits	n=46	n=23 (FCVB + silicone oil)	Device ‐ FCVB	‐ FCVB to replace the natural vitreous body in the 14 treatment of severe RD in both rabbits and Human. ‐ It was made of silicone rubber and consisted of a thin vitreous‐shaped capsule with a tube‐valve system. The capsule was fabricated via a computer simulation of the human and rabbit vitreous cavities and the use of a drainage tube connected to a drainage valve. The medium can be injected into the capsule via the tube‐valve system to maintain suitable intraocular pressure and support the retina. The FCVB can provide 360‐degree arc support for the retina via its analogous solid strength, which thus provides a new mode of treatment for retinal detachment. When the capsular membrane restored the appearance of the natural vitreous, it segregated the medium from the retina so as to prevent the intraocular toxicity that would be caused by direct contact with the retina and the inordinate flow caused by current vitreous substitutes.	Control (contralateral eye)	Mainly RD	
Self‐assembling hydrogel loaded with 5‐FU PLGA microspheres as a novel vitreous substitute for proliferative vitreoretinopathy	Yu, Z. and Ma, S. and Wu, M. and Cui, H. and Wu, R. and Chen, S. and Xu, C. and Lu, X. and Feng, S.	To use PVA/chitosan hydrogel as a novel in situ crosslinked vitreous substitute and 5‐FU‐loaded PLGA microspheres as an accessory treatment for PVR. The feasibility of this composition was evaluated in comparison with human vitreous, and its safety was examined in cell‐based studies. Also, we evaluated its long‐term effect, and biocompatibility in the retina via implanting it into the rabbit vitreous cavity.	Rabbits	N/A	n=12	Hydrogel	PVA/chitosan hydrogel as a novel in situ crosslinked vitreous substitute and 5‐FU‐loaded PLGA microspheres as an accessory treatment for PVR.	Human vitreous	N/A	
Study on the effectiveness and safety of Foldable Capsular Vitreous Body implantation	Zhang, X. and Tian, X. and Zhang, B. and Guo, L. and Li, X. and Jia, Y.	To evaluate the efficacy and safety of the implantation of foldable capsular vitreous body in 1‐year follow‐up.	Human	n=20	n=20	Device ‐ FCVB	FCVB was designed to support the retina combined with more than 5000 cSt SO injected through the drain tube, which is a brand‐new therapy strategy. FCVB implantation had the aid of the surface tension of SO, not only 360° supported the retina, but also isolated the SO with the capsule of FCVB. By extracting or supplying SO through the drainage valve of the FCVB, the IOP can be regulated. Since that, the key problem of simply SO tamponade was solved, no more emulsification of SO would occur under the protection of FCVB. Further‐ more, patients did not need to maintain prone posture after surgery. Made from medical grade biocompatible polymer (FDA registered material), the FCVB can directly contact with the tissue in the eyeball and barely induce irritation. Moreover, in the rabbit model of PVR, SO‐injected FCVB closely fitted the inner structure of eyeballs and restored basic features (support, refraction and cellular barriers) without causing any complication after im‐ plantation. In addition, in vitro studies, no significant changes of genes or proteins of retinal pigment epithe‐ lium were detected after FCVB was implanted into eye‐ ball.	N/A	Several; including RD, ocular rupture, vitreous hemorrhage, ocular atrophy after SO tamponade, CR detachment, recurrent RD, SO dependent eye, intraocular lens implantation. There were 13 cases of eyeball rupture, 5 cases of SO dependent eyes, one case of endophthalmitis, and one case of eyeball atrophy.	
Dual‐Crosslinked Betaine‐Based Amphiphilic Hydrogel as a Promising Vitreous Substitute: Anti‐Adhesion, Anti‐Fouling, and Anti‐Cell Proliferation	Cai Y, Tan Y, Li Y, Zhang Z, Qin J, Chen H, Li C, Zhou L, Liu T, Zhou Y	To characterize a dual‐crosslinked betaine‐based amphiphilic polymer hydrogel (BAPH) with self‐healing, anti‐cell adhesion, and anti‐fouling properties as a long‐term vitreous substitute, and to evaluate its biocompatibility, optical stability, and functional tolerance in vitrectomized rabbit eyes over 90 days	Rabbits	n=36	n=12	Hydrogel	A betaine‐based amphiphilic copolymer hydrogel [P(SBMA‐co‐AANa)] synthesized by in‐situ copolymerization of sulfobetaine methacrylate (SBMA) and acrylic acid (AA) with poly(ethylene glycol) diacrylate (PEGDA‐1000) as a trace chemical crosslinker. The optimized composition (BAPC0.8) forms a dual network of covalent and ionic crosslinks.	Silicone oil and BSS	N/A	

**Study**	**Study Type (e.g Retrospective, Prospective, RCT, etc)**	**Surgical Methods (e.g. 24g PPV)**	**Results**							
			**Volume Injected (e.g. cc)**	**Success Rate (%) (e.g. tamponade of the retina)**	**Anatomical/Surgical Outcomes**	**Physical Properties**	**Clinical Outcomes**	**Biochemical Outcomes**	**Optical Outcomes (Refractive Index and Light Transmission)**	**Other Outcomes/Notes**

Stable oxime‐crosslinked hyaluronan‐based hydrogel as a biomimetic vitreous substitute	Prospective	23G Total Plus Vitrectomy Pak	1 cc	Not reported	The rabbit retina remained healthy and intact over the course of the 56 day histology. The retina did not become detached with the HA‐oxime hydrogel and the structure remained intact.	The density of the HA‐oxime hydrogel is 1.01 ± 0.05 g mL− 1 (similar to the native vitreous). The refractive indices of the HA‐oxime hydrogel (1.36 ± 0.002) is comparable to the native vitreous. The surface tension is significantly higher than that of silicone oil, so it will not migrate into retinal tears or anterior chamber. HA oxime gel degraded by day 56.	Mean intraocular pressures were all reported within normal range for rabbits (13.5‐25.92).	The number of nuclei in the GCL, INL and ONL remained similar across all timepoints, demonstrating that the HA‐oxime hydrogel is biocompatible.	Refractive Index: 1.36	Key to the success is the stable click chemistry.
Vitreous Substitutes Following Vitrectomy Surgery	Phase 1 Clinical Trial	Vitrectomy (details not reported)	Not reported		Vitargus was confirmed to fill the vitreous cavity.	Vitargus is an injectable, transparent, oxihyaluronic acid‐adipic acid dihydrazide hydrogel, transmitting all wavelengths of visible light. It has a refractive index of 1.34, close to that of human vitreous (1.33)	Found the mean BCVA improved after surgery by 31.9 ± 32.8, 21.4 ± 44.0, 31.9 ± 32.8 letters (mean ± SD) at day 1, day 7, and month 1 respectively (P < .05), compared to 16.5 ± 21.2 letters at baseline. Three patients experienced elevated IOP: one requiring laser and trabeculectomy, one underwent implantation of drainage tube and one with elevated IOP and appearance of sterile hypopyon.	No apparent toxicity to ocular tissues.	Refractive Index: 1.34	Vitargus sets as a stable semisolid gel adhering to the retina and maintains its position without the need for face down positioning.
Design of an injectable in situ gelation biomaterials for vitreous substitute	Prospective	PPV with two port systems: 21G at 2.5 mm from the limbus. The vitreous body was removed as much as possible and replaced with air. Then, each sample was injected into the vitreous cavity. Two port wounds were closed with 8‐0 polypropylene suture and treated with cryopexy (NO2) for 7 s. After surgery, the eyes were treated with Levofloxacin as antibiotic eyedrops and betamethasone sodium phosphate as steroid eyedrops three times a day for 1 week.	Not reported	100% initially. No follow‐up reported.	‐ E10KDC18 polymer solution exhibits rapid gelation in the vitreous cavity and does not leak out of the cavity. ‐ E10KDC18 gel exerts a hydraulic pressure and sufficient rigidity to provide tamponade to the retina against the pigment epithelium layer	‐ Transmission of light (>90%) similar to natural vitreous to reach the sensory elements at the back of the eye.	‐ No marked adverse reactions (elevated IOP, inflammation) were detected ‐ Polymer able to be injected through small gauge needles (e.g 21G)	‐ Chemical inertness before and after injection into vitreous	‐ Refractive index (1.353) similar to natural vitreous (1.336)	‐ No loss of retinal tissue was observed by histopathologic examination of retinal layers
Safety of medium‐chain triglycerides used as an intraocular tamponading agent in an experimental vitrectomy model rabbit	Prospective	20G PPV	0.8‐1.5 cc	71%	inferior RDs in 8 eyes, secondary to a retinal break probably during vitrectomy ‐ in all of these cases superior retina remained attached ‐MCT misinjected into anterior chamber of one eye (this was excluded) ‐2 other eyes: vitreous cutter touched the lens during operative procedure, causing posterior subcapsular opacity P6	‐The density of the triglycerides at 20°C was 0.94 to 0.95 g/cm^3 for a viscosity at 20°C of 27 to 33 mPa·s, and the refractive index was 1.449 to 1.451 ‐Solution of MCT was homogenous and stable in presence of water, air, and perfluorocar‐ bon liquid (PFCL).	‐Histologic examination did not reveal any retinal toxicity. ‐Two cases of moderate emulsification were observed, but in these cases, emulsification was caused by the perioperative injection of the agent. ‐13 cases of inflammatory reaction in vitreous cavity and no case of inflammatory reaction in anterior chamber. ‐Two eyes developed cataract as a result of perioperative trauma to the lens with the vitreous cutter and not secondary to the presence of medium‐chain triglycerides in the vitreous cavity.	N/A	‐Refractive index was 1.449 to 1.451.	
A cross‐linked hyaluronic acid hydrogel (Healaflow) as a novel vitreous substitute	Prospective	Combined 25‐20G PPV + PVD	1 cc	83%	‐Success rate was found to be 100% at the time of euthanization (42 to 105 days post‐op), with the exception of 2 eyes that experienced iatrogenic partial retinal detachment, and 2 eyes developed significant cataracts from intraoperative complications. One rabbit was lost due to unrelated reasons.	‐Maintained viscous structure in the cavity for at least 2 weeks. ‐Estimated specific gravity is circa 1.03.	‐In two eyes significant cataract developed due to intra‐operative complications. ‐Postoperative IOP (15–25 mmHg) was slightly elevated compared to earlier results.	Routine microscopy and immunohistochemistry demonstrated normal morphology with some Müller cell activation (upregulation of glial acidic fibrillary protein, GFAP) compared to unoperated eyes and no significant DNA‐ fragmentation (TUNEL‐assay).	Refractive index i = 1.341.	‐The gel appeared to maintain its viscous structure in the vitreous cavity for at least 2 weeks, potentially allowing for an effective short‐term tamponade. ‐The fact that the iatrogenic retinal detachments were self‐limiting on long‐term follow up may support the notion of a tamponading effect
Perfluoro‐n‐octane as a temporary intraocular tamponade in a staged approach to manage complex retinal detachments	Retrospective	23G PPV	Not reported	94%	‐ 16/17 eyes (94%) had complete reattachment ‐ 1/17 partial reattachments ‐ 0 re‐detachments occurred	N/A	All eyes retained or improved visual acuity. ‐ Two eyes had long‐term intraocular pressure of 5 mmHg and no eye had intraocular pressure of >21 mmHg. No long‐term inflammation was observed. ‐ Three eyes (all three eyes initially had globe ruptures with loss of intraocular tissue and loss of the crystalline lens) developed a hazy cornea during the follow‐up period; none of these eyes had a permanent endotamponade with silicone oil ‐no endophthalmitis	N/A	N/A	N/A
New biodegradable networks of poly(N‐vinylpyrrolidinone) designed for controlled nonburst degradation in the vitreous body	Prospective	‐ Implantation of the dry cylindrical specimens (2‐3 mm length, 0.8 mm diameter) ′‐ Incision in pars plana with sclerotomy knife (no mention of PPV, nor needle gauge) ‐ Test specimen placed with hollow needle + inserted into the vitreous at the incision point	N/A	N/A	‐ Smooth and reproducible swelling and degradation in the absence of a burst effect	‐ Material A dissolves and degrades by approximately 70% during the first week	‐ Implantation of these materials did not cause any harmful biocompatibility effects, such as inflammation, irritation or infection ‐ However swelling of the large cylindrical objects was a risk for cataract formation (seen in material B)	‐ There was absolutely no cellular reaction to the implants	‐material A was found to dissolve and degrade by approximately 70% within 1 week and optically changed from transparent to opaque white within several hours in vitro, but returned to transparent; ‐material B exhibited faster swelling and degradation with dissolution within 1 day; ‐materials C and D dissolved shortly after incubation; ‐material E demonstrated similar characteristics to material A; ‐material F was similar to material B; ‐material G was akin to materials C and D.	
Functional evaluation of a novel vitreous substitute using polyethylene glycol sols injected into a foldable capsular vitreous body	Prospective	Standard three‐port PPV	1.0‐1.2 mL	100% at 3 months	‐ capsule of FCVB fitted well with the shape of the vitreous cavity. Also, B‐scan ultrasonography showed that FCVB enhanced echoes in the epiretinal membrane, prompting FCVB to support retinal attachment via a mechanical pressing method, and have an all‐round retina supporting function ‐ no retinal detachments were observed	‐ In the FCVB‐implanted eyes, PEG sols appeared homogeneous and transparent on the inside, except for the precipitates that existed in the inferior and posterior wall of the capsule ‐ In vitro analysis indicated that viscosity in PEG‐implanted eyes at days 30, 90, and 180 was at 17.7, 5.8, and 3.9% of the original value, respectively ‐ In the FCVB‐implanted eyes, viscosity was 72.0, 65.7, and 56.8% of the original value.	‐ There was a slight conjunctival hyperemia by day 7 in all groups ‐ Lens subluxation (n=2) in FCVB‐ implanted eyes ‐ The incidence of cataract varied in different groups at days 30, 90, and 180, respectively. In the FCVB‐implanted eyes, the incidence of cataract was 25% (2/12), 66.7% (6/9), and 100% (4/4, n ¼ 2 for lensectomy). ‐ In the PEG‐implanted eyes, the incidence of cataract was 11.1% (1/9), 33.3% (2/6), and 33.3% (1/3). – In the BSS‐implanted eyes, the incidence of cataract was 11.1%(1/9) ‐IOPs returned to baseline within 14 days	N/A	A concentration of 5% (w/v) of the PEG sols showed that pH at 7.12, density at 1.049, interfacial tension at 62.107 dyn/ cm, refractive index at 1.336 nD, light transmittance at 80.48%, and viscosity at 1787.9 cP, whose properties were most similar to natural vitreous, and was chosen as the vitreous substitute in in vivo tests.	Clinical and pathological examinations showed that FCVB injected with PEG sols had good biocompatibility in the rabbit eyes as no corneal opacity, intraocular inflammation, vitreous hemorrhage, and retina detachment were observed, with the exception of a higher incidence of cataracts and lens subluxation. The application of the FCVB can prolong the retention time of the PEG sols.
Clinical device‐related article evaluation of morphology and functions of a foldable capsular vitreous body in the rabbit eye	Prospective	Three‐port PPV (with sclerotomy 4.0 mm posterior to the limbus)	Not reported	The success rate was found to be 100% over the months for the FCVB eyes vs in the silicone oil group, 50% (5/10) retinal detachments reoccurred.	‐ FCVB supported the retina and eye well by providing a solid arc, while silicone oil was unable to adequately support the retina or eye by interfacial surface tension alone ‐ It can be made very fine and thin, and can be folded and inserted via a very small incision to support differently shaped eyes. Its thin (30 um) and flexible nature allows FCVB implantation through a small surgical incision. ‐ Since the FCVB implantation does not require a routine fluid‐air exchange it may reduce surgical complications of PPV	N/A	‐ In FCVB group 2/10 animals developed cataracts, in contrast to 5/10 rabbits with silicone oil ‐ No complications such as intraocular inflammation, retinal hemorrhage or detachment were observed in the FCVB group, in contrast in 5/10 retinal detachments recurrent in silicone oil filled PVR eyes ‐ No difference in IOP between two groups	N/A	‐ Three months after PPV, a significant hyperopic shift of 2.00 6 1.17 D was observed in the silicone oil‐treated group (p = 0.029). ‐ In the FCVB group, the average refractive change was þ0.20 6 0.45 D, and the difference was not significant (p = 0.73).	If FCVB were applied clinically to the human eyes, the refractive outcome would be expected to result in emmetropia.
Reattachment after foldable capsular vitreous body implantation in severe retinal detachment eyes	Retrospective	23G PPV	Not reported	The retinal reattachment rate was 92.59%, as observed via fundus photography, OCT scans, B‐scans, or computed tomography scans.	‐ FCVBS were positioned to support the retina well and the retinal reattachment rate reached 92.59% ‐ One of the patients had a balloon break during surgery and a drainage tube exposure after surgery. However, no other severe complications including endophthalmitis, sympathetic ophthalmia and rejection of FCVB associated with FCVB were detected		‐ There was a statistically significant difference between preoperative and postoperative visions ‐ The postoperative IOP (7.93 ± 3.57 mm Hg) was significantly lower than the preoperative IOP (13.98 ± 10.72 mm Hg) ‐ Of the 27 cases, 14 exhibited corneal opacity without keratopathy, and 13 exhibited a shallow anterior chamber. ‐In five cases, postoperative bleeding occurred in early surgical patients	N/A	The corneal transparency of the normal anterior chamber (mean rank 20.04) was significantly higher than that of the shallow anterior chamber (mean rank 7.50, *χ*2 = 22.42, P = 0.00).	
A novel vitreous substitute of using a foldable capsular vitreous body injected with polyvinylalcohol hydrogel	Prospective	20G PPV.	1.1 cc (PVA alone), 1.4 cc (PVA+FCVB)	100% at POD180	FCVB injected with PVA hydrogel as a vitreous substitute had good biocompatibility and retina support function in the vitreous cavity after a long‐term (180 days) tamponade.	Appreciable biodegradation of the substitute in the PVA group and a decrease of viscoelastic properties after 180 days of tamponade in vivo. Conversely, in the PVA + FCVB group, the PVA retained its rheological features without biodegradation at 180 day follow‐up.	‐ Anterior chamber inflammation was visible in all groups post‐operatively, but was more severe in the PVA + FCVB group and only recovered within seven days with intensive anti‐inflammatory treatments. ‐ Cataracts: In the BSS group 17% eyes developed cataracts, in the PVA group 50% eyes developed cataracts. PVA+FCVB group had lensectomy during PPV to circumvent second operation. ‐ No evidence of vitritis, uveitis, retinitis, endophthalmitis, vitreous hemorrhage, or retina detachment in the rabbit eyes of all groups ‐ No statistically significant differences were found in the IOP among the three groups preoperatively or at 7, 14, 30, 60, and 90 days postoperatively, but a significant difference was found after 180 days: The IOP of the PVA group was significantly decreased at 180 days (potentially due to the fact that some of the PVA hydrogel was dissolved in the vitreous cavities)	No toxic reactions seen.	‐ 1% PVA: RI 1.3355, LT 94% ‐ 3% PVA: RI 1.3361, LT 93% ‐ 7% PVA: RI 1.3425, LT 88%	
Evaluation of a viscoelastic solution of hydroxypropyl methylcellulose as a potential vitreous substitute	Prospective	A gas vitrectomy with PFP was performed with a 30G needle.	0.5 mL	Not reported	‐ Immediately after HPMC injection the eyes were hypotonic, and when injected into the vitreous cavity, it was not detected in the aqueous humor ‐ 28 hours later the aqueous humor gave a positive reaction for HPMC ‐ Low concentration of HPMC seen in the vitreous cavity of the eye with a large retinal detachment ‐ possibly due to the breakdown of the diffusion barrier of the normal retina ‐ Maintenance of retinal pigment epithelial cell characteristics	‐ The viscosity dropped nearly one half its room temperature value when it reached body temperature ‐ Viscosity will drop to only 200 mPa.s upon being diluted in half the intraocular fluids ‐ Because HPMC solutions are diluted by the intraocular fluids, they do not have an interfacial tension against the intraocular fluids + will not seal retinal holes in rhegmatogenous detachments	‐ 1/2 of the six‐week rabbits had a 180 degree retinal detachment and the injected eyes of the other rabbits had no remarkable differences among them ‐ Absence of HPMC toxicity ‐ No cell death or proliferation documented	‐ Elimination of HPMC from the vitreous cavity of the rabbit seems to be through the aqueous humor and Schlemm′s canal	Not reported	‐ unlikely that HPMC solution will be useful as a long‐term vitreous replacement when at least three months or longer are needed for the viscous vitreous substitute supporting the retina during the restoration of a chorioretinal adhesion ‐ however, a viscoelastic solution of HPMC might be useful in the short term (for removing intraocular silicone oil, peeling membranes and to avoid postoperative hypotony)
A new strategy to replace the natural vitreous by a novel capsular artificial vitreous body with pressure‐control valve	Prospective	Standard three‐port PPV, with sclerotomies 2.5‐mm posterior to the limbus			‐ The capsule of the novel vitreous with PBS was in good contact with the inner retina and can support it well ‐ Good tissue integrity with no structural abnormality such as deformations, degeneration or inflammation ‐ No structural damages in other parts of the eye (including cornea and ciliary body) ‐ It was easy to implant through a 1.5 mm scleral injection	‐ Absorbance values obtained did not show a significant difference compared to control	‐ Slight conjunctival hyperaemia by day 7 of surgery ‐ Cataracts in 2/20 rabbits ‐ Otherwise: no serious complications including corneal opacity, intraocular inflammation, and retinal haemorrhage or detachment, were observed over 8 weeks ‐ the tonometric measurements showed no significant difference of IOP between the two groups	‐ Good biocompatibility (skin irritation test, sensitization tests, febrile responses, acute systemic toxicity, subcutaneous implantation test and haemolysis) according to ISO guidelines	‐ Transmittance > 93%	N/A
Effect on rabbits′ intraocular structure by cross‐linked hyaluronic formations as vitreous substitute	Retrospective	20G subtotal vitrectomy	1.0‐1.2 cc	83% at 42 days	‐UV‐CHA biogel remained stable for > 6wk in the vitreous cavity of rabbits. ‐During the whole observation period, the color of the optic disc and the retinal blood vessels of the different concentrations of the experiment were normal. No disc edema, optic atrophy, retinal necrosis, and no retinal tissue were observed.	‐Kinematic viscosity is much higher (61‐94 Pa·s) than that of silicone oil (approximately 4.85 Pa·s) which has a dynamic viscosity of 5000 cSt and remains higher in kinematic viscosity at low shear rates.	‐No cataracts occurred ‐IOP increased in the first week after surgery. In research group, comparing with the first week after surgery, the IOP in HA 24 mg/mL and 40 mg/mL groups decreased gradually. At week 4, the IOP was close to the normal control group (P>0.05).	‐At least 6 weeks of stability of HA gel were seen in the vitreous cavity ‐Good biocompatibility of the cross‐linked sodium hyaluronate gel	‐This study suggests that cross‐linked sodium hyaluronate gel appears to have good transparency in the vitreous cavity for at least 6wk. ‐The refractive index of UV‐CHA hydrogel is similar to human vitreous body or water, although the specific value was not reported.	
Perfluorohexylethan (O62) as ocular endotamponade in complex vitreoretinal surgery	Prospective Clinical Study	PPV performed on all patients (no details re PVR given)	Not reported	100% initially ‐ difficulties in 45.5% of eyes	‐ O62 was able to flatten and reposition the central retina. However, differences in physical properties of O62 compared with perfluorocarbon liquids (PFCLs) lead to difficulties in reattaching the peripheral retina ‐ The intraoperative and initial post‐operative retinal attachment rate was 100%. In 7 of 11 eyes, the retina remained attached during the O62 tamponade and after its removal ‐ Recurrent retinal detachments with PVR developed in 4 of 11 eyes under the tamponade, of which 2 were located superiorly 1 was inferior, and 1 was total. Two of four redetachments associated with PVR developed in eyes with intentional incomplete filling of the vitreous cavity with O62.	‐ O62 is a low viscosity substance	‐ Preoperative median visual acuity was 20/400. Due to severe O62 emulsifications and penetration of several bubbles into the anterior chamber and on the surface of the posterior lens capsule, the postoperative median visual acuity under O62 endotamponade decreased to 20/800. Final median VA at last follow‐up and after cataract extraction was 20/100 ‐ In all eyes, a marked inflammatory reaction in the originally not filled anterior segment with fibrin formation and cell flare was observed in the early postoperative period, which responded well to topical corticosteroid medication ‐ IOP was increased in 2/11 patients ‐ Starting in the second week, severe emulsification of O62 was observed in all eyes	‐ toxic reaction to the partially fluorinated alkanes	Optical outcomes reported were a refractive index of 1.29, and a viscosity of 0.75 mPa·s, similar to that of well‐known PFCLs.	‐ due to its extreme and early emulsification and the unclear inflammatory reactions, O62 is not suitable as a long‐term ocular endotamponade
Long‐Term Biocompatibility of a Highly Viscously Thiol‐Modified Cross‐Linked Hyaluronate as a Novel Vitreous Body Substitute	Retrospective	20/23G PPV with PVD	Vitreous substitute was added until egress was appreciated from the second sclerotomy site.	78% (if plausible iatrogenic RDs are included)	‐No changes or differences in the integrity, attachment and thickness of the retina at any time point	Physicochemical characterization of the naturally cross‐linked gel revealed a refractive index similar to the human vitreous, and the transparent, hydrophilic material was shown to be highly elastic but still easily injectable and comparable shear moduli to other vitreous substitutes.	‐22% rabbits developed RD, 75% of which occurred within 1 month after surgery, and 25% occurred between 1 and 3 months after surgery. ‐28% of rabbits developed iatrogenic cataracts, of which 40% occurred within 1 month after surgery, and 60% occurred between 1 and 3 months after surgery. 22% rabbits developed RD, 75% of which occurred within 1 month after surgery, and 25% occurred between 1 and 3 months after surgery. ‐No signs of infection ‐IOP never reached worrisome high or low levels at any point after surgery	‐Stability persisted over a long period of time ‐ No toxic reactions besides expected fibrin reaction of anterior chamber after surgery	‐Excellent refractive index of 1.338 ‐Cornea stayed clear in all rabbits throughout study ‐Lens also stayed clear besides in cases of iatrogenic lens touch	
The feasibility study of an in situ marine polysaccharide‐based hydrogel as the vitreous substitute	Prospective	PPV (gauge not specified)	Not reported	100% at 5, 30 and 90 days	‐100% success rate ‐Rapid formation of a space‐filling, transparent hydrogel in vitreous cavity ‐Combined rod‐cone maximal response in the operated eyes were significantly reduced compared with those in normal rabbit eyes, suggesting a decline of vision.	‐Hydrogel has high elasticity and a certain viscosity and might be suitable for vitreous substitution.	‐Minor decline of vision and decrease in densities of cones and rods in the operated rabbit eyes were observed. ‐No significant inflammation or any other adverse reactions were identified in anterior chamber of the operated eyes. ‐No complications, such as hemorrhage from vitreous body, retinal detachment, or choroidal detachment, were identified in the operated eyes of tested rabbits ‐The IOPs of the operated eyes at POD2, 4, 6, and 8 were significantly decreased (P<.05), while the IOPs obtained thereafter were all similar to the preoperative IOPs.	N/A	‐Cornea and lens were clear and maintained transparency during the 90‐day follow‐up. ‐Small amount of tiny white opacity was observed in the central vitreous 30 days after surgery which disappeared in the following months ‐Refractive index (i = 1.3348), transmittance (>80%), pH (7.45 ± 0.04) and density (1.015 ± 0.052 g/cm^3) of the hydrogel were comparable to natural human vitreous.	
Application of thermo‐setting gel as artificial vitreous	Prospective	After conjunctival incision, 20G PP sclerotomy was carried out at two sites. After the vitreous was excised, 1 mL of WTG was injected by a 27G blunt needle.	1 cc	100%	‐In the presence of a retinal tear, the injected WTG drifted under the detached retina through the retinal tear before gelation occurred. This phenomenon indicates the possibility that the WTG gel will drift under the retina and remain there. ‐This implies that the WTG injection may not be expected to provide a tamponade effect, which is an important role of an ocular filling substance, to fill the vitreous cavity and facilitate reattachment of the retina. ‐No structural changes in the ciliary body, anterior chamber angle, lens, or cornea. ‐Despite having been subjected to surgical invasion, the operated eyes showed no difference from the contralateral control eyes.	‐Low viscosity made handling and injection very easy. ‐After injection, it transforms to gel form at body temperature.	‐No IOP difference. ‐Slight conj hyperemia on POD1 & 3 ‐Otherwise, no abnormal findings, including corneal opacity, inflammation in anterior chamber, lens opacity, vitreous opacity, retinal hemorrhage, or retinal detachment, were observed during the whole course of observation.	‐WTG induced no abnormal biological reactions ‐WTG is hydrophilic and may be discharged together with the aqueous humor through Schlemm’s canal.	‐Retains transparency even upon gelation. ‐RI not reported. ‐LT 89.3%. ‐Adjusting the gel’s composition can alter its viscosity, light transmission, and gelation time.	‐Since outcomes were only evaluated up to 28 days, it might not be adequate to evaluate biological compatibility and prolonged observership for safety is needed.
Foldable Capsular Vitreous Body Implantation for Complicated Retinal Detachment Caused by Severe Ocular Trauma	prospective, single‐arm, surgical interventional case series study.	23G PPV	Not reported	100% of cases	The retina was reattached in all cases during the operation, and the FCVB was successfully implanted in the vitreous cavity in all cases.		‐ Most patients maintained preop vision after surgery. Postop BCVA improved in 7 cases compared with preop, BCVA remained unchanged in 21 cases. No statistically significant difference between postop and preop BCVA (P < 0.05). ‐ Mean IOP 7.01 ± 2.43 mmHg before surgery and 8.54 ± 2.93 mmHg after surgery, and the difference was statistically significant (P < 0.05). During FU period of all cases, FCVB was intact and well positioned, retina was attached, shape of eyeball in all cases was maintained well, and the appearance did not seem to be significantly different from the contralateral eye. ‐ Conj / scleral hyperemia in all cases disappeared between 1 and 2 months after surgery and did not affect appearance eventually. Some patients had a mild FBS within 1 month after surgery. No atrophy of eyeballs occurred. Postop satisfaction was significantly better than preop satisfaction (P < 0.05). No pts regretted decision.		The optical properties of FCVB indicate that transmittances are 92%, hazes are 5.74%, and spectral transmittance is 97%, which reveal that FCVB is a highly transparent material and can meet the requirement of an artificial vitreous body to be transparent.	
Evaluation of the flexibility, efficacy, and safety of a foldable capsular vitreous body in the treatment of severe retinal detachment	Prospective	PPV, and MP, retinotomy, and relaxing retinectomy were added if necessary.	4.0 mL into FCVB	Retinal reattachment was found in 8 (73%) of 11 eyes at the end of the 3‐month treatment time.	‐ Retinal reattachments in 73% of eyes at end of 3‐month treatment time. ‐ Fundus of FCVB‐filled eyes was clearly visible and the FCVB was well distributed in the vitreous cavity at 3 days. ‐ Retina was reattached and capsule of FCVB supported it and whole eye well, without any wrinkles forming in retina during the 3‐month implantation period. ‐ OCT indicated the 60um‐thick capsular membrane evenly supported retina. ‐ Retinal reattachments were observed in 8 and in 5 of 8 eyes at 1 and 3 months after FCVB removal, respectively.		‐ IOP at 3‐month implantation time was lower than at baseline. However, IOPs at each time point in FCVB‐treated eyes did not show a significant difference, except at 4 weeks (P < 0.015) during the 3‐month implantation and 3 months after capsule removal (P < 0.05). ‐ Scores for VA at each time point did not show a significant difference from those at baseline (P < 0.05, Fig. 3B) ‐ Slight conj hyperemia by POD7 in FCVB‐filled eyes. ‐ No serious complications (e.g., corneal keratopathy or intraocular inflammation) observed. ‐ No statistically significant conj congestion, corneal edema, keratic precipitate, or aqueous flare at baseline or at 3 months s/p implantation. ‐ In 2 cases, anterior chamber hyphema was observed.		FCVB changes the refraction very little when compared with silicone oil and heavy silicone oil.	
Retinal‐detachment repair and vitreous‐like‐body reformation via a thermogelling polymer endotamponade	Prospective	23G PPV	1.15‐1.5 cc	100% (7 days)	OCT mac at 3 and 12 months revealed normal architecture with the formation of adequate chorioretinal adhesion at the site of iatrogenic retinal breaks.	‐Gelation temp decreases from 27.7 °C to 12.3 °C, over a range of 3 to 12 wt% EPC concentrations. ‐EPC 3% was able to attain a surface tension of at least 40mN m^‐1 at both 24 and 37 degrees celsius, well above that of silicone oil. ‐Swelling counterforce of the solutions increases with temperature.	‐ 75% EPC‐12% rabbits had raised IOP of ≥30 mmHg at day 14. In 2 of 3 rabbits, IOP was normal (<30 mmHg) by day 30. ‐EPC‐3% and EPC‐7% hydrogels showed no significant inflammation in either anterior nor posterior segments, and had normal IOP. ‐EPC hydrogels remained optically clear in the vitreous cavity with attached retina. However, rabbits implanted with EPC‐12% hydrogel developed severe sub‐acute inflammation by 2 weeks post‐surgery associated with elevated IOP.	Consistent with OCT, reduction in both inner and outer retinal layer thickness only in EPC‐3%‐filled eyes.	‐Transparency at body temperature (37°C) and a refractive index of 1.339 to 1.344 (similar to that of native vitreous, 1.337) due to high (>90%) water content.	
Evaluation of collagen gel and hyaluronic acid as vitreous substitutes	Prospective	Bimanual vitrectomy was done through the PP using Ocutome and a fiberoptic endoilluminator. Vitrectomy, followed by injection of 0.5 ml of FITC‐collagen. FL‐HA, the mixture, or BSS (Alcon).	0.5 mL	Not reported	Upon injection of FITC‐collagen into vitreous cavity following vitrectomy, collagen accumulated on cavity bottom and was observed to be in direct contact with internal limiting membrane of retina.		‐ SLE: no study eyes had marked damage anteriorly, except for a slight reaction caused by the surgery. Ctrl eyes showed the same minor reactions. ‐ IOP varied among the normal rabbits from 15‐18 mm Hg before injection of HA, the collagen gel, the mixed gel, and BSS. ‐ Statistical analysis revealed slight increase in IOP at 1 and 3 days post‐op. ‐ No marked IOP increase was observed at 7, 14, 30, and 60 days post‐op.	‐ LM of retina 1 month after injection showed no marked retinal abnormalities, and retinal architecture was well preserved. ‐ EM showed that neither the internal limiting membrane nor the Müller fibers exhibited any marked abnormalities.	Optical outcomes demonstrated that FITC maintained its transparency during the 3‐month period.	‐ ERGs showed no marked differences between control eyes and eyes injected with vitreous substitutes. ‐ Fluorophotometry: FL‐HA had shortest life in vitreous cavity followed by FITC‐collagen and the mixed gel. The median values of the longevity of FITC‐ collagen, and the mixed gel were 2.4 and 3.2 months, respectively. ‐ FITC‐collagen and FL‐HA did not cause any ocular tissue damage, because the microstructures of these tissues were well preserved for 3 months compared with controls.
Super‐fast in situ formation of hydrogels based on multi‐arm functional polyethylene glycols as endotamponade substitutes	Prospective	25G PPV (no triamcinolone)	0.8‐1.2 cc	100% (6 months)	According to FFA and SD‐OCT, the retinal breaks healed well and the retinal photocoagulation was clear.	‐ The constructed hydrogels were all very close to those of human vitreous humor in physical features and parameters, e.g., viscosity.	‐Anterior segment: slight inflammation at 1 and 7 days after surgery in BSS, AVB2 and AVB3 groups, which were comparable to the normal control. ‐Inflammation reaction found in high‐concentration AVB4 group at 1 day post‐op, and the phenomenon lasted 7 days. ‐Severe inflammation or even endophthalmitis occurred in the BSS group, as reported previously. ‐A decrease in IOP was seen in the first 3 days after surgery in all operated groups, possibly due to the post‐surgery one‐week reduction of IOP caused by the unhealed scleral puncture	‐Compared with the BSS group and normal ctrl eyes, the AVB2 and AVB3 groups had no disorganized microstructure, apparent inflammation cells, hemorrhage, or edema at one month after implantation in the rabbits’ eyes, while in the AVB4 group, chronic inflammation cells infiltrated the retina and optic disk, along with degeneration in the retinal nerve fiber layer. ‐No inflammation cells were found in the ciliary bodies in all sections.	‐Excellent transparency was seen when applying the hydrogels, and their light transmittance was all above 90% (AVB1 (95.3%) 4 AVB2 (94.8%) 4 AVB3 (93.0%) 4 AVB4 (91.0%) at 37°C at a wavelength of 550 nm. ‐Refractive index was 1.3364‐1.3390	‐ AVB2 groups had transparent cornea and lens, and obvious red‐reflex after implanting AVB in the rabbits’ eyes for 3 and 6 months, in contrast to non‐operated control group and the BSS group. ‐ Hydrogel adhered to the retina tightly, and no changes in the retinal structure and no tissue damage, edema, hemorrhage or subretinal fluid were found except in the AVB4 group. Moreover, the retinal barrier function was intact, with no fluorescence leakage during the early (within 1 min) or late (after 5 min) phases of FFA at 3 and 6 months after implanting AVB2.
Experimental vitreous replacement with perfluorophenanthrene	Prospective	21G PPV	0.8‐1.0 mL	Not reported			‐ After surgery, posterior capsular cataract developed in 4/24 of the eyes. ‐ RD was found at POW3 in 1 of the 4 eyes where PFP had been removed from the vitreous cavity after one week. ‐ No changes in IOP were present in any of the eyes.	Histo/immunohisto: ‐ After 2 hours: no histological alterations ‐ After 7 days: migration of phagocytes up to ILM; edema of the IPL; early detection of ganglion cells with pyknotic nuclei ‐ After 15 days: disorganization of the layer of nuclei and lack of ganglion cells; PFP droplets below the ILM; increase in the histological findings observed after seven days ‐ After 30 days: phagocytes with inclusion of large PFP droplets; increase of ganglion cell pyknosis ‐ After 60 days: fibroblasts, neutrophilic granulocytes, giant cells; histiocytic inflammation with fibrosis; marked changes to the retinal architecture. ‐ In the group of 4 eyes where PFP was removed from the vitreous cavity 7 days after surgery and the eyes were enucleated after one month, the histological picture did not show any regression of the retinal lesions observed in another group of rabbits after seven days of PFP in the vitreous cavity.	Not reported	
Bioinspired Fibrillary Hydrogel with Controlled Swelling Behavior: Applicability as an Artificial Vitreous	Prospective	23G PPV	0.5‐0.6 cc	100% at 30 days	OCT was performed on 4 rabbits in each group and showed no RD 30 days post‐op.	‐Thiolated gellan hydrogels had negligible swelling in 1× PBS, with a normalized DoS nearly equivalent to 1, in contrast to the thiolated poly[methacrylamide‐co‐(methacrylic acid)], which were highly swellable (normalized DoS ∼ 2). On the other hand, the two‐component hydrogels swelled in 1× PBS had a normalized DoS (normalized DoS ∼ 1.6) lower than that of the copolymer, CoP, and higher than that of thiolated gellan, G. ‐The swelling pressures produced by both the hydrogels are comparable to that of the overall eye pressure (11−21 mmHg) ‐Viscosity not recorded.	‐No significant inflammation in the anterior segment of the eye on POD 1, 7, and 30. ‐The conjunctiva was red on POD 1, but this redness was significantly reduced on POD 4, and the conjunctiva was clear and colorless by POD7. ‐The lenses were clear, and the Y‐suture was visible in rabbits treated with the hydrogels. ‐60% of silicone‐oil‐treated (n = 10), 27% of 0.9G_12CoP‐treated (n = 11), and 55% of 1.5G_10CoP‐treated (n = 11) rabbits had a slight posterior polar cataract at POD 30 . Specifically, in 3 of the 6 1.5G_10CoP hydrogel‐treated rabbits, the cataract was highly localized at the surgical entrance of vitrectomy. ‐Presurgical and postsurgical IOP measurements were comparable for all experimental groups. ‐Preoperative and PO IOPs were comparable for all experimental groups. The 0.9G 12CoP group had a significant decrease in IOP on POD1 (significance level not reported), but this was resolved by postoperative day 4. The immediate postoperative IOPs for all experimental groups were slightly higher than reported rabbits IOPs in the literature, the values were in an acceptable range for the postoperative time period, and the increased IOP subsided by POD1.		‐Similar to natural vitreous ‐RI and LT not reported	
Efficacy of two different thiol‐modified crosslinked hyaluronate formulations as vitreous replacement compared to silicone oil in a model of retinal detachment	Prospective	23G PPV	N/A	81%	‐88% of rabbits that received silicone oil developed a partial (1 animal) or total RD (six animals) with pronounced PVR within the first 2 weeks after surgery. ‐In contrast, in the VBS strong group only 38% rabbits developed a new RD, 67% of which were were only partial detachments. ‐No recurrence of RD was observed in the VBS soft group.	‐The generated VBS both revealed a refractive index of 1.34 at 589 nm and 1.32 at 546 nm, respectively. ‐Rheological analysis with hydrogel pre‐sheared by ejection from a 5 mL syringe through a 20g needle resulted for VBS soft in a storage modulus (G’; *ω* = 1 1/s) of 150400 mPa and a loss modulus (G”; *ω* = 1 1/s) of 4693 mPa. ‐Injection force, a parameter reflective of viscosity, of the hydrogel was 5.3N for VBS soft and 14.2N for VBS strong, respectively. This force is comparable to the force needed to inject ophthalmic viscoelastic devices during cataract surgery. In short, all physical properties were similar to normal vitreous.	‐IOP and retinal morphology were normal as long as the retina remained re‐attached. ‐IOP after silicone oil filling was significantly lower compared to the contralateral eye throughout the entire follow‐up period, whereas in both hydrogel groups the IOP returned to pre‐op levels at the end of the study. ‐Only a minority of eyes which received VBS soft (29%) or VBS strong (13%) developed cataracts. One of the cataracts in the VBS soft group was most likely due to iatrogenic lens touch during surgery. ‐Majority of silicone oil filled eyes (88%) had developed dense cataracts at the one month visit. None of the cataracts in the silicone oil group was due to iatrogenic factors.		Optically, tVBS both revealed a refractive index of 1.34 at 589 nm and 1.32 at 546 nm, respectively. LT not reported.	‐In the majority of VBS soft or strong eyes OCT was feasible and showed attached and normally structured retina. ‐It could be shown that the data of the ERG measurements were normally distributed, which was important for the selection of the appropriate statistical tests for the evaluation of the ERG results. ‐The ERG of the silicone oil group was unchanged in only one rabbit, one was strongly reduced and in six rabbits the ERG was completely extinguished.
The cross‐linked biopolymer hyaluronic acid as an artificial vitreous substitute	Prospective	20G PPV	N/A	67%	‐After PPV the UV‐CHA hydrogels were easily injected via the pars plana into the vitreous cavity of the rabbit eye. However, in 33% of eyes, iatrogenic RD occurred during the preceding vitrectomy. Nevertheless, these eyes were also filled with UV‐CHA and included in the follow‐up. ‐Interestingly, the retina remained attached in 75% of cases with iatrogenic RDs although the RDs were not specifically treated.	Rheological assessment suggested adequate viscosity and elasticity for intraocular use.	‐ SLE and funduscopy: no signs of inflammation or disintegration of the hydrogels was visible. ‐ IOP was normal during the entire follow‐up.	Good biocompatibility was further confirmed by histology	‐ Cross‐linked hydrogels consistently revealed a refractive index of 1.338 in all experimental series. This result is almost identical with that in human vitreous (1.336) or water.	‐ The cross‐linked hydrogels remained stable over months. Consistency, transparency, and 3‐d structure were maintained throughout. ‐ The transparency and viscoelasticity of the injected UV‐CHA was grossly unchanged since injection 6 weeks earlier.
Outcomes of surgery for retinal detachment associated with proliferative vitreoretinopathy using perfluoro‐n‐octane: A multicenter study	Prospective, noncomparative, interventional multicenter study.	PPV	Not reported	77% at one year	‐ A relaxing retinotomy was performed in 231 (42%) eyes, and intraoperative retinal slippage was noted in 10 (2%) of these eyes. ‐ Iatrogenic retinal breaks were reported in 90 (16%) patients. ‐ Complete retinal reattachment was achieved intraoperatively in 507 (91%) eyes.		‐ BCVA of 20/200 or better was measured in 51 (10%) patients preop and 85 (25%) patients at 6 months PO. Compared with preop, postop VA improved in 274 (60%) eyes, remained stable in 106 (23%), and worsened in 85 (18%) eyes. Of the 300 patients with VA of 5/200 or less preop, 56 (18%) improved to 20/200 or better at 6 months postop. ‐ Six‐month FU was obtained for 356 (65%) eyes; the retina was attached in 279 (78%) eyes, retained PFO was noted in 20 (6%), and the number of eyes with corneal edema, elevated IOP, and hypotony were 26 (7%), 6 (2%), and 48 (15%), respectively. These rates are similar to those of the last follow‐up rates in the entire group. ‐ Of the 114 phakic eyes without significant cataract preop, 105 (92%) developed a significant cataract or underwent cataract surgery during the study FU interval. ‐ Of the 121 phakic eyes with cataract preop, 103 (85.2%) underwent CE during study follow‐up. Retained PFO was noted at any time during follow‐up in 41 (7.4%) eyes; retained PFO was not a significant predictor of either recurrent RD or visual outcome.			
Rabbit study of an in situ forming hydrogel vitreous substitute	Prospective cohort	25G PPV	N/A	90%	The success rate was found to be 100% at 7 days, excluding one iatrogenic RD.	The hydrogel had viscoelastic properties akin to natural vitreous.	‐SLE: no significant inflammation of anterior segment at examinations on POD 1 and 7. Trace cells were seen in all surgical eyes on POD 1, which resolved by examination on day 7. In 2 of the rabbits, a vacuole was present in the anterior vitreous cavity that appeared to be consistent with an air bubble. ‐On digital palpation of globes, none of the rabbits presented with unusually firm eyes at the examinations on either 1 or 7 days PO. In fact, the right eyes felt comparable to their counterpart left eyes. Serial funduscopic examination of tested eyes revealed no evidence of vitritis, uveitis, retinitis, or endophthalmitis. ‐1 rabbit demonstrated a retinal hole which was iatrogenic in nature and developed into a localized detachment by the examination on POD7. In the remainder of the specimens, there was no evidence of RD or SRF.	Examination of H&E stained retinal sections under LM revealed that the integrity of the retinal layers and RPE appeared preserved with no evidence of toxicity or vacuolization. A few specimens demonstrated inflammatory cells localized only to the preretinal space, primarily near the vitrectomy site with some spillover posteriorly. Sections of the right (experimental) eyes that received the 2% hydrogel vitreous substitute were otherwise identical by LM to those of the respective left eyes.	‐RI of 1.33. ‐LT not reported.	
F6H8 as an intraoperative tool and F6H8/silicone oil as a postoperative tamponade in inferior retinal detachment with inferior PVR	Retrospective case series	PPV 10 patients were not operated on for RD before undergoing PPV surgery with F6H8. Of the 12 patients who had previous vitreoretinal surgery, 8 underwent PPV with scleral buckle (SB), 2 underwent PPV without SB, and 2 underwent SB without PPV.	Not reported	95.45%	‐ F6H8 was efficacious as an intraoperative agent used to flatten the retina in all cases. ‐ Regarding retinal behavior during the presence of the F6H8/SO tamponade, we observed retinal attachment without progression of PVR in 16 out of 22 patients (72%). ‐ Among the remaining patients, we observed recurrent inferior PVR in 2 (ratios of 70/30 and 60/40, resp.) and recurrent tractional RD with superior PVR in 3 (all with 30/70 ratio).	Perfluorohexyloctane has the following physical properties: density of 1.3 g/cm3, viscosity of 2.5mPa⋅s, and an interface tension against water of 49.1 mN/m.	‐ During FU, 1 pt with a F6H8/SO ratio of 70/30 showed increased IOP the day after surgery, which was controlled with medications for the next 20 days, until it returned to be WNL. ‐ 5 patients, with ratios of 60/40 (3), 50/50 (2), and 30/70 (1), showed low IOP, which remained in the low range throughout the follow‐up period, without any clinical signs of hypotony. ‐ During FU period posterior synechiae were observed in 5 patients: 1 with a F6H8/SO ratio of 70/30, 1 with 60/40, 1 with 60/40 in the first operation and 50/50 in the second operation, and 2 with 30/70. ‐ In 1 patient inferior retinal hemorrhages were observed but were resolved one month after surgery (40/60 ratio). ‐ 2 patients, both with a ratio of 30/70, developed CME, as determined by OCT. ‐ 1 patient with 50/50 ratio a migration of the tamponade into the anterior chamber occurred through a partial inferior zonular dehiscence, forming bubbles of the F6H8/SO mixture in the anterior chamber but not causing corneal damage. ‐ No patient showed emulsification or signs of vasculitis or uveitis.		density of 1.3 g/cm3 and a transparent mixture present with all ratios besides 70/30 and 60/40.	‐ Due to her compromised general conditions 1 patient was followed up for 14 months without F6H8/SO removal: no signs of inflammation or uveitis were observed, except for the formation of mild posterior synechiae; the retina remained attached although a pucker was observed.
A self‐assembling peptide gel as a vitreous substitute: A rabbit study	Prospective	25G PPV	N/A	100%	No posterior pole abnormalities at any follow‐ups.	‐ The substitute had a storage modulus (G’) that was higher than the loss modulus (G”) at all frequencies. This indicated that the solution exhibited a typical “gel‐like” behavior. It had a G’ of 18.12 6 2.00 Pa at 1 rad/s.	‐ SLE revealed no significant inflammation, hemorrhage, or other disease on POD 1, 3, 7, 14, 21, 28 (1 month), 56 (2 months), and 84 (3 months). ‐ Additionally, no rabbits developed cataracts during the follow‐up period. ‐ Fundoscopic examination also revealed no abnormal findings in the optic nerve or ocular fundus. The vitreous substitute remained transparent, with no opacities observed. ‐ IOP remained WNL and was not significantly different between the control and vitreous substitute groups at any time point examined.	There was no difference in the fluorescence staining between the RPE cells incubated in the vitreous substitute for 24 hours and the negative controls. In contrast, nearly all RPE cells incubated with Triton X‐100 exhibited a red fluorescence stain. The proliferation of RPE cells in the vitreous substitute group was not significantly different from that in the control group (P > 0.05).	‐ Refractive index of the vitreous substitute was 1.3339 and the visible light transmission rate was 96.7%.	‐ No significant abnormalities in both the control and vitreous substitute groups were observed on the ERGs. ‐ The a‐ and b‐wave amplitudes were comparable between groups and remained WNL during the entire follow‐up period. ‐ The postop implicit times were not significantly different from the preoperative times in either group at any time point examined.
Antifouling Super Water Absorbent Supramolecular Polymer Hydrogel as an Artificial Vitreous Body	Prospective	22G PPV	0.5‐1 cc	100% at 6 weeks	‐ B‐scan test examined retinal integrity. No evident echoes were observed in the normal, sham‐operated and PNAGA‐PCBAA‐10‐4 groups. While for the PNAGA hydrogel group, there were echoes of the unidentified object, which could be originated from RD or intraocular foreign body reaction elicited by this pristine gel. ‐ For normal, sham‐operated and PNAGA‐PCBAA‐10‐4 hydrogel group, no vitritis, uveitis, retinitis, endophthalmitis, vitreous hemorrhage, or retinal detachment was observed in the rabbit eyes.	‐ Overall, the values of G ^′^ and G ^′′^ gradually decreased with increasing temperature. Comparatively, the G ^′^ and G ^′′^ values of PNAGA‐PCBAA‐20‐4 hydrogel were higher than those of other two hydrogels due to the stronger hydrogen bonding interaction in the copolymer network at a higher mon‐ omer content. ‐ The mechanical properties of a hydrogel are important for fulfilling its supporting function. Next, the frequency sweep mode of rheological measurement was performed on the PNAGA‐PCBAA‐b‐4 (b = 10, 15, and 20) hydrogels. Generally speaking, G ^′^ was larger than G ^′′^ at all frequencies from 0.01 to 10 Hz, and there was no crossover, suggesting a “gel‐like” behavior.	IOPs of the sham‐operated group and PNAGA were higher than that of normal group. Particularly, the PNAGA hydrogel exhibited a much higher intraocular pressure.		PNAGA hydrogels have 94.7% water content, 1.3572 RI, but as low as 53.3% and 51.1% light transparency before and 6 months postimplantation, respectively, an indication of translucent property.	
Biocompatibility and retinal support of a foldable capsular vitreous body injected with saline or silicone oil implanted in rabbit eyes	Prospective	Standard PPV	1.1 mL	100% from FCVB	‐ SLE: no abnormal events such as silicone oil emulsification, retinal hyperaemia or optic atrophy in the experimental groups for 180 days. ‐ The fundus was clear and the capsular wall of the FCVB fit perfectly with the retina in all quadrants. However, many silicone oil vesicles were found on the posterior surface of the lens in all eyes (n = 5) of the silicone tamponade group on the first day after surgery, and the number of silicone oil vesicles increased and existed in the vitreous cavity during subsequent observations.		‐ Among the 3 groups, there was no other obvious inflammatory reaction or other disease (keratopathy or shallow anterior chamber) except cataracts, which emerged in the anterior segment of the eyes over 180 days. ‐ No statistically significant differences were found among the three groups in the IOP preop or at 1, 3, 7, 14 and 28 days, but they were found at 56, 90 and 180 days postoperatively. However, these differences were in the normal range at each time point.	‐ In the histological examination, the layers of cornea epithelial cells and the epithelial cells of the ciliary body were intact, without a loss of normal ocular tissue and with full thickness of the retina. ‐ There was no evidence of pathological changes in any ocular tissues or structural abnormalities such as deformations, degeneration or inflammation in either of the two experimental groups. However, the silicone oil tamponade alone group showed many vacuoles in and disorganization of the ciliary body.	In the rabbit PVR model, we found that the FCVB with a balanced salt solution (BSS) very closely mimicked the morphology and restored physiological functions such as support, refraction and cellular barriers during a 3‐month observation period, without silicone oil complications.22 In addition, FCVBs injected with BSS changed refraction less than silicone oil or heavy silicone oil tamponade	Scan found some mild opacity echoes in the vitreous cavity and a smoothly increased epiretinal echogenicity in all FCVB injected with saline rabbit eyes (n = 9), which appeared to be the posterior wall of the FCVB. But there was interference of the silicone oil in the FCVB injected with silicone oil eyes during the 180 days.
Outcomes of a Foldable Capsular Vitreous Body Implantation: A Retrospective Study	Retrospective	PPV	Not reported	100% at 12 months	During FU, no FCVB rejection, displacement, rupture, exposure, sympathetic ophthalmitis, bullae keratopathy, or other serious surgical complications were observed in any patient.		‐ Vision. 12 eyes had no light sensation before surgery, 3 eyes had preserved light sensation, and 3 eyes had manual sensation. 12 months PO, 8 eyes had no light sensation, 3 eyes had preserved light sensation, and 7 eyes had manual sensation. Among them, there were 7 eyes with improved vision, 10 eyes without obvious improvement, and 1 eye with decreased vision. ‐ IOP. Preop IOP was in the range of 3.00–30.00 mmHg in all subjects. The IOP was in the range of 3.00–26.00mmHg 1 week after surgery, which represented a significant change from baseline (t = −0:66, P = 0:52). 12 months after surgery, mean IOP of the other 16 eyes was 10:13 ± 3:52 mmHg, except for 2 eyes in which IOP could not be measured due to corneal degeneration. The IOP of 6 eyes was of <8.00 mmHg and that of 12 eyes was in the normal range (8.00–21.00 mmHg). No patient had IOP of >22.00 mmHg, and these values did not change from baseline (t = 0:38, P = 0:71).		Not reported	
Feasibility study of chitosan as intravitreous tamponade material	Prospective cohort	PPV	Between 1.2 and 1.5 mL	100%			‐ No significant difference in IOP at different time points between the experimental group and the control group (all P>0.05). ‐ In the experimental group, the IOP POD1 was no different from that before the operation (P=0.08), and this was also the case in the control group (P=0.126), indicating that the surgical operation itself didn’t cause obvious fluctuation of IOP.	‐ Concentration of IL‐6, IL‐8 and NO in aqueous humor. At POD15, the concentration of IL‐6 in aqueous humor in the experimental and control groups were significantly higher than in the blank control group, but there was no statistically significant difference b/w the experimental group and the control group. There was no significant difference in the concentration of IL‐8 and NO in aqueous humor b/w the experimental and control groups and the blank control group. At POD30, the concentration of IL‐6, IL‐8 and NO showed no significant difference among the three groups. ‐ Concentration of IL‐6, IL‐8 and NO in vitreous body. At day 30 post‐op, there was no significant difference in the concentrations of IL‐6 in vitreous body among the three groups. The concentrations of IL‐8 and NO in the vitreous body in the experimental and control groups were higher than in the blank control group (all P < 0.05), but there was no significant difference between the experimental group and the control group. ‐ Similar to the blank control group, the cornea epithelial cells in the experimental group and the control group were intact and well‐arranged, the fibrous structure of stroma layer was identical, Bowman’s membrane and Descemet’s membrane was intact, and the endothelial cells were regular and their size was the same. The muscular layer, stromal layer and epithelial layer of ciliary body were normal in the three groups. The posterior lens capsule was intact in the 3 groups and no pathological changes were observed.	Optical outcomes reported that the cornea, lens and vitreous cavity remained transparent throughout the entire study.	
Preliminary study on retinal vascular and oxygen‐related changes after long‐term silicone oil and foldable capsular vitreous body tamponade	Prospective	20G PPV	1.2 mL	Not reported			‐ SLE. Anterior chamber inflammation was almost visible in both treated groups in the early postop period and a few rabbits in the FCVB 1 silicone oil group had relatively severe cases, with some inflammatory fibrinous exudation in the anterior chamber. How‐ ever, they recovered within POW1 when given anti‐inflammatory treatments. ‐ No serious complications, such as keratopathy, posterior synechia or iris neovascularization observed over 180 days. ‐ IOPs of the silicone oil and FCVB 1 silicone oil groups maintained relatively gentle fluctuation within the normal range during the postop observational period, and regarding the IOPs, no statistically significant differences were found between the two treated groups and contralateral control eyes, either preoperatively or at 7, 14, 30, 60, 90 and 180 days postoperatively (p . 0.05). ‐ Fundus and fluorescein angiography. no abnormalities, such as choroiditis, retinal hemorrhage, retinal hole or retinal detachment, were discovered in the two treated groups.	‐ Expression of total retinal HIF‐1a and VEGF mRNA. No significant variations were observed in HIF‐1a mRNA at 30, 90 and 180 days postop in the silicone oil and FCVB1 silicone oil groups compared to the contralateral control eyes. ‐ VEGF protein concentrations, as detected by ELISA. The normal‐ ized VEGF protein concentrations in the retinas at 30, 90 and 180 days postoperatively. No significant differences were noted at 30, 90 and 180 days postoperatively in the silicone oil group as compared to the contralateral eyes. ‐ Histopathologic findings. There was no evidence of pathological changes in retinal tissues or structural abnormalities, such as deformations, degeneration or inflammation, in either of the two groups. Moreover, there were no pathological alterations in other parts of the eye, including the cornea and ciliary body. ‐ Immunohistochemical staining analysis. To investigate the spatial and temporal expressions of HIF‐1a and VEGF in the retina, we performed immunohistochemical staining on retinal cross‐sections at 90 and 180 days postop.	All eyes showed clarity in the vitreous cavities, although no other optical outcomes were reported.	
Self‐assembling hydrogel loaded with 5‐FU PLGA microspheres as a novel vitreous substitute for proliferative vitreoretinopathy	Prospective	25G PPV	0.9 cc	N/A	No success rate was reported.	At all frequencies, for all PVA/chitosan hydrogel concentrations, G′ was higher than G", and the preparation showed gelatinous characteristics. The curves of G′ and G" were almost parallel and there was no intersection.	‐No complications, such as corneal opacity, keratopathy, nor posterior synechiae, were observed within 24 weeks. The experimental groups had a significantly increased IOP at post op week 1 which decreased IOP by 4 months (P<0.05). ‐At week 1, the IOP of PVA and PVA/MS group showed an upward trend, which was significantly different from the Control group (∗p < .05). At weeks 4, IOP decreased and stabilized from 9 to 10 mmHg after weeks 8. From weeks 16, the IOPs in the PVA and the PVA/MS group were significantly lower than those in the Control group (∗p < .05).	′‐ There was a strip of hyperechoic pre‐retina in the Control group and the PVA group 24 weeks after the operation, suggesting that the slit‐lamp photographs at Week 4, 12, and 24. White arrows point to opacity in the vitreousfibrous proliferative membrane reappeared. There was no significant hyperechoic in PVA/MS group, suggesting no proliferation ‐ In the Control group, the epiretinal membrane is thickening at Week 24. Also, the retinal detachment and RPE cells proliferation were observed. In the PVA group, the epiretinal membrane is slightly thickening with no retinal detachment. In the PVA/MS group, no epiretinal membrane thickening, retinal detachment, or RPE cells proliferation was observed	Refractive index of the substitute ranged from 1.33 ‐1.62, with a light transmittance from 83 to 93%.	
Study on the effectiveness and safety of Foldable Capsular Vitreous Body implantation	Retrospective	PPV	Not reported	30% achieved re‐attachment (6/20 eyes)	FCVB well supported the vitreous retina in all treated eyes, and 6 treated eyes achieved retinal reattachment 12 months after FCVB implantation.	N/A	‐ No change in VA. Before operation, VAs were unsatisfactory, 55% of the patients under NLP condition. Only 1 patient’s VA had regression from finger count in 30 m scopes (FC/30 cm) to light perception or hand moving (LP‐HM), while there were no significant changes in the VA of the other patients before and after the operation (P = 1.000), ‐ Improved IOP. Contrast to the preop IOP was 12.90 ± 7.06 mmHg, the postoperative IOP elevated to 15.15 ± 3.36 mmHg. According to the results of IOP records, it was normal in 1 cases, low in 17 cases and high in 2 cases before operation, while 10 cases showed low IOPs, and the IOP of other 10 cases returned to normal level in 12 months after the operation. The difference between preoperative and postoperative IOPs was statistically significant. ‐ 12 months after FCVB implantation, B‐ultrasound was conducted and 6 of the 20 patients developed retinal reattachment, while the retina of the other 14 was lost or damaged, due to severe eye damage.	The implanted FCVB can sustainably and mechanically release dexamethasone sodium phosphate (the molecular mass = 516.41 Da) through the apertures in a time‐dependent and a dose‐dependent manner	Not reported	No severe complications associated with FCVB were detected, though some patients suffered mild hemorrhage and omental proliferation caused by previ‐ ous severe ocular damage.
Dual‐Crosslinked Betaine‐Based Amphiphilic Hydrogel as a Promising Vitreous Substitute: Anti‐Adhesion, Anti‐Fouling, and Anti‐Cell Proliferation	Prospective	25G PPV with PVD, no induced RD	1.0–1.5 mL per eye	NA (no RD model)	The retina remained attached and morphologically intact over the 90‐day follow‐up. OCT and B‐scan ultrasonography confirmed a clear vitreous cavity without inflammation or opacity. Retinal and choroidal thicknesses showed no statistical difference from BSS or SO groups on days 15, 30, or 90. H&E histology at day 30 showed normal layered retinal structure with no inflammatory infiltration or necrosis; heart, liver, and kidney also appeared normal. OCTA revealed no microcirculatory abnormalities at days 15, 30, or 90	BAPC0.8 displayed G ^′^ (elastic modulus) 100–500 Pa > G″ (viscous modulus), comparable to native vitreous. Viscosity–shear rate profile showed shear‐thinning behavior similar to rabbit vitreous. Refractive index was 1.341–1.390 (close to native vitreous) with a density of ~1.01 g·mL⁻¹. The hydrogel showed self‐healing, injectability, and slow degradation >30 days in vivo.	Intraocular pressure (IOP) transiently decreased to 10.2 ± 1.1 mmHg at day 3 and normalized (17.0 ± 1.1 mmHg) by day 7. All IOP values remained within 11–21 mmHg thereafter. No cataracts, inflammation, corneal edema, or vitreous haze were observed up to 90 days. ERG analysis showed stable a‐ and b‐wave amplitudes across 90 days with no significant deviation from baseline.	The hydrogel exhibited low protein adsorption (≈ 0.24 *μ*g·cm⁻²) and low ARPE‐19 adhesion, with viability > 98% at 24 h. Wound‐healing assays demonstrated suppressed RPE migration (p < 0.01). Aqueous interleukin levels (IL‐1*β*, IL‐6, TNF‐*α*) at day 30 were not significantly different from BSS or SO groups (Figure S21). Histopathology of heart, liver, and kidney confirmed systemic safety.	Transparency: 80–95% across 350–800 nm, >90% above 500 nm	Dual chemical and ionic crosslinking, providing stable elasticity, high transparency, and biocompatibility. Its anti‐fouling and anti‐cell adhesion properties, matched viscoelasticity and refractive index, and optical stability for ≥ 90 days make it a strong candidate for a long‐term vitreous substitute.

**Table 2:** Recent studies (last 3 years) investigating experimental vitreous substitutes with small sample sizes (<10 eyes).

**Study**	**Authors**	**Aim**	**Study Subjects (e.g. Human, Monkey, Rabbits, etc)**	**Total Eyes # (n=)**	**Eyes that received Experimental Subject # (n=)**	**Type (Device, Hydrogel, Polymer, Smart Gel, etc)**	**Substitute Description**	**Comparator Substitute (if any)**	**Disease studied (retinal detachment, endophthalmitis, proliferative vitreoretinopathy, etc)**	

Injectable alginate‐based in situ self‐healable transparent hydrogel as a vitreous substitute with a tamponading function	Choi G, An SH, Choi J‐W, Rho MS, Park WC, Jeong W, Cha HJ	To develop and evaluate a transparent alginate‐phenylboronic acid/polyvinyl alcohol (TALPPH) composite hydrogel as a novel vitreous tamponade compared with silicone oil and room air.	Rabbits	n=26	n=8	Hydrogel	A transparent, self‐healable hydrogel composed of alginate functionalized with phenylboronic acid (PBA) dynamically crosslinked with poly(vinyl alcohol) (PVA) through reversible boronate–diol bonds. The composite, termed TALPPH, exhibits rapid in‐situ gelation, shear‐thinning injectability, and viscoelastic behavior mimicking the native vitreous.	Silicone oil and room air	Retinal detachment	
Evaluating the feasibility and safety of a self‐crosslinked hyaluronic acid hydrogel for experimental retinal detachment repair	Yang X, Meng J, Kang Y, Yin Z, Lu J, Xu J, Zhang Y	To assess safety and efficacy of a biocompatible self‐crosslinked HA hydrogel for retinal reattachment vs silicone oil and balanced salt solution (BSS).	Rabbits	n=15	n=6	Hydrogel	A self‐crosslinking hyaluronic acid (HA) hydrogel generated by mixing aldehyde‐modified HA (HA‐CHO) with amine‐modified HA (HA‐NH₂) to form in situ imine (Schiff‐base) linkages under physiological conditions. The resulting hydrogel is fully transparent, biodegradable within 14 days, and maintains the viscoelasticity and optical properties of native vitreous.	Silicone Oil and BSS	Retinal detachment	
In Vivo Assessment of an Antioxidant Hydrogel Vitreous Substitute	Allyn MM, Ryan AK, Rivera G, Mamo E, Bopp J, Martinez Hernandez S, Racine J, Miller EJ, Chandler HL, Swindle‐Reilly KE	To evaluate the biocompatibility, tolerability, and antioxidant performance of a PEGDA hydrogel vitreous substitute loaded with ascorbic acid and glutathione following vitrectomy in rabbits compared to silicone oil and sham controls.	Rabbits	n=21	n=7	Hydrogel	A poly(ethylene glycol) diacrylate (PEGDA) hydrogel containing ascorbic acid (AA) and glutathione (GSH) as antioxidant additives. The hydrogel was synthesized via acrylate polymerization, producing an injectable, transparent, and viscoelastic network with mechanical properties similar to native vitreous.	Silicone Oil	None	

**Study**	**Study Type (e.g Retrospective, Prospective, RCT, etc)**	**Surgical Methods (e.g. 24G PPV)**	**Results**							
			**Volume Injected (e.g. cc)**	**Success Rate (%) (e.g. tamponade of the retina)**	**Anatomical/Surgical Outcomes**	**Physical Properties**	**Clinical Outcomes**	**Biochemical Outcomes**	**Optical Outcomes (Refractive Index and Light Transmission)**	**Other Outcomes/Notes**

Injectable alginate‐based in situ self‐healable transparent hydrogel as a vitreous substitute with a tamponading function	Prospective	23G PPV with PVD and induced RD	Not reported (filled vitreous cavity)	75% (6/8 eyes) retinal reattachment vs 57% (SO) and 33% (room air)	None of the groups showed any fibrin formation, endophthalmitis, or other posterior inflammatory reactions post‐operatively at the last time point of examination. Histological evaluation revealed intact retinal layers with normal ganglion cell and photoreceptor morphology in the TALPPH‐treated eyes, comparable to unoperated controls. No cellular infiltration, fibrosis, or gliosis was observed.	The viscosity (~25 000 mPa·s) was substantially higher than that of the native vitreous (300–2000 mPa·s) and silicone oil (~900 mPa·s), providing stronger tamponade capability. The surface tension was 65 mN m⁻¹. The gel demonstrated shear‐thinning behavior, rapid in‐situ gelation, and self‐healing properties.	Mean intraocular pressures were all reported within the normal range for rabbits (13.5–25.9 mmHg) throughout the 3‐month postoperative period. No significant postoperative inflammation, corneal opacity, or endophthalmitis was observed in the TALPPH group. In contrast, eyes filled with silicone oil showed a transient anterior chamber fibrin reaction at 1 week that resolved spontaneously.	The number of nuclei in the ganglion cell layer (GCL), inner nuclear layer (INL), and outer nuclear layer (ONL) remained similar across all time points. Immunohistochemical analysis showed minimal expression of glial fibrillary acidic protein (GFAP) in Müller cells, indicating limited gliosis. No inflammatory cell infiltration or fibrotic encapsulation was observed around the retinal tissue. In vitro, human retinal pigment epithelial (ARPE‐19) cells cultured with TALPPH exhibited high viability and low cytotoxicity.	The TALPPH hydrogel exhibited a density and refractive index (1.336–1.342) comparable to the native vitreous. The optical transparency ranged from 82–91%, maintaining an average of 87 % at baseline, 84 % after 1 month, and 82 % after 7 months, indicating long‐term optical stability.	Hydrogel is self‐healing viscoelastic network formed by reversible boronate–diol crosslinks between alginate‐PBA and PVA
Evaluating the feasibility and safety of a self‐crosslinked hyaluronic acid hydrogel for experimental retinal detachment repair	Prospective	23G PPV with PVD and induced RD	1.3 ±0.2 mL for the HA group, 1.5 ±0.1 mL for the SO group, and 1.4 ±0.1 mL for the BSS group	100 % (6/6 eyes) retinal reattachment vs 67 % (4/6 SO) and 0 % (0/3 BSS)	The rabbit retina remained healthy and intact over the course of the 14‐day follow‐up. Complete retinal reattachment was achieved in 6 of 6 eyes (100%) in the HA hydrogel group. OCT and fundus imaging confirmed flat retinas with normal contour in HA‐treated eyes, while partial detachment persisted in the silicone oil group and total detachment in BSS eyes. Histological sections revealed preserved retinal layers and no signs of photoreceptor loss or gliosis in the HA group, indicating strong tamponade and minimal inflammatory response.	The in‐situ self‐crosslinked HA hydrogel had a pH of 7.20 ±0.05, an osmotic pressure of 313 ±8 mOsm/kg and a density of 1.025 ±0.009 g/cm3. The degradation period was approximately 14 days, during which the material gradually resorbed and was replaced by aqueous humor, leaving the vitreous cavity optically clear. The gel is formed within seconds after mixing aldehyde‐modified HA (HA‐CHO) with amine‐modified HA (HA‐NH₂).	On postoperative day 1, the IOP in the HA hydrogel group decreased to 6.9 ± 1.9 mmHg, then rose to 10.2 ± 1.1 mmHg by day 3, returning to baseline (17.0 ± 1.1 mmHg) after 1 week. There was no significant difference in IOP between the hydrogel‐ and silicone oil–treated eyes (p > 0.05). A mild anterior chamber inflammatory response was noted during the first 3 days following surgery, which resolved completely by day 7. No secondary cataracts were observed in any group throughout the study period.	Histological analysis showed well‐preserved retinal architecture with clearly delineated layers (ONL, INL, and GCL) in the HA‐treated eyes, similar to those of unoperated controls. In contrast, silicone oil–treated retinas displayed partial thinning of the photoreceptor layer, and BSS eyes showed disorganization and folding of the retina. GFAP immunostaining revealed minimal Müller glial activation in the HA group, while the SO and BSS groups exhibited strong GFAP expression, indicating reactive gliosis secondary to retinal stress. TUNEL staining demonstrated significantly fewer apoptotic nuclei in the HA‐treated retinas compared to SO and BSS groups, confirming that the hydrogel minimized retinal cell apoptosis following detachment and repair.	The hydrogel has a refractive index of ~1.34, comparable to the native vitreous. The hydrogel is transparent and viscoelastic (G ^′^ > G″).	In‐situ crosslinking between aldehyde‐ and amine‐modified HA chains, which created a biodegradable hydrogel capable of providing stable retinal tamponade without chronic inflammation
In Vivo Assessment of an Antioxidant Hydrogel Vitreous Substitute	Prospective	25G PPV with PVD, no induced RD	Not reported (filled vitreous cavity)	NA (no RD model)	The retina and optic nerve remained healthy and structurally intact throughout the 28‐day follow‐up. Retinal thickness was unchanged relative to control eyes. Minimal conjunctival edema was observed postoperatively across groups, resolving spontaneously. One silicone oil (SO) eye developed a focal anterior subcapsular cataract, attributed to surgical manipulation.	The PEGDA hydrogel exhibited a storage modulus greater than the loss modulus (G ^′^ > G″) within the linear viscoelastic region (<10% strain), indicating predominantly elastic behavior. The gel was slightly stiffer than native vitreous and retained its shape following injection.	Mean intraocular pressures averaged 18.2 ± 3.4 mmHg over 28 days. IOP remained stable across all time points and did not differ significantly between groups after day 7. No adverse ocular events were observed in the hydrogel group. One eye in the silicone oil group developed a cataract. SPOTS scoring showed clear optical media, absence of inflammation, and no vitreal haze. Electroretinography (ERG) demonstrated no significant changes in a‐ or b‐wave amplitudes or implicit times, confirming preserved retinal function.	Lens ascorbic acid and glutathione concentrations were significantly higher in the hydrogel group compared with controls (p < 0.05), reflecting sustained antioxidant release. Catalase activity was elevated in both hydrogel and SO groups (p = 0.0453) relative to controls. No differences in GSH/GSSG ratio or total aqueous protein were observed, indicating absence of oxidative stress or inflammation. Histopathology confirmed normal retinal and lens morphology with minimal immune cell infiltration.	It remained optically transparent throughout the study period without syneresis or phase separation.	Controlled release of ascorbic acid and glutathione, maintaining intraocular antioxidant defenses post‐vitrectomy

### 3.3. Risk of Bias Assessment

The SYRCLE Risk of Bias Assessment tool for animal studies was used to assess all animal studies (*n* = 28; Table 3). The SYRCYLE tool showed wide heterogeneity in the design of animal studies. Only 6/28 animal studies controlled for group characteristics to be similar at baseline and adjusted for confounders in the analysis. One out of twenty‐eight animal studies concealed group allocation. Three out of twenty‐eight animal studies utilized random animal housing during the experiment. One out of twenty‐eight animal studies had the caregivers and/or investigators blinded from knowledge of which intervention each animal received during the experiment. Two out of twenty‐eight animal studies selected animals at random for outcome assessment. One out of twenty‐eight study utilized a blinded assessor for outcome.

**Table 3:** Risk of bias assessment of included animal studies using the SYRCLE Risk of Bias tool.

**Study**	**Authors**	**SYRCLE TOOL (Yes, No, or Unknown)**									

		**Was the allocation sequence adequately generated and applied?**	**Were the groups similar at baseline or were they adjusted for confounders in the analysis?**	**Was the allocation adequately concealed?**	**Were the animals randomly housed during the experiment?**	**Were the caregivers and/or investigators blinded from knowledge which intervention each animal received during the experiment?**	**Were animals selected at random for outcome assessment?**	**Was the outcome assessor blinded?**	**Were incomplete outcome data adequately addressed?**	**Are reports of the study free of selective outcome reporting?**	**Was the study apparently free of other problems that could result in high risk of bias?**

Design of an injectable in situ gelation biomaterials for vitreous substitute	Annaka, M. and Mortensen, K. and Vigild, M. E. and Matsuura, T. and Tsuji, S. and Ueda, T. and Tsujinaka, H.	No	No	No	No	No	No	No	Unknown	No	No

Safety of medium‐chain triglycerides used as an intraocular tamponading agent in an experimental vitrectomy model rabbit	Auriol, S. and Mahieu, L. and Brousset, P. and Malecaze, F. and Mathis, V.	No	Yes	No	No	No	Yes	No	Yes	Yes	Yes

A cross‐linked hyaluronic acid hydrogel (Healaflow) as a novel vitreous substitute	Barth, H. and Crafoord, S. and Andr√©asson, S. and Ghosh, F.	No	No	No	No	No	No	Unknown	Yes	Yes	Yes

New biodegradable networks of poly(N‐vinylpyrrolidinone) designed for controlled nonburst degradation in the vitreous body	Bruining, M. J. and Edelbroek‐Hoogendoorn, P. S. and Blaauwgeers, H. G. and Mooy, C. M. and Hendrikse, F. H. and Koole, L. H.	No	No	No	Unknown	No	No	No	Yes	Yes	Yes

Functional evaluation of a novel vitreous substitute using polyethylene glycol sols injected into a foldable capsular vitreous body	Chen, H. and Feng, S. and Liu, Y. and Huang, Z. and Sun, X. and Zhou, L. and Lu, X. and Gao, Q.	Yes	Yes	Unknown	Yes	No	No	No	Yes	Yes	Yes

Clinical device‐related article evaluation of morphology and functions of a foldable capsular vitreous body in the rabbit eye	Chen, J. and Gao, Q. and Liu, Y. and Ge, J. and Cao, X. and Luo, Y. and Huang, D. and Zhou, G. and Lin, S. and Lin, J. and To, C. H. and Siu, A. W.	Yes	Yes	Unknown	Yes	No	No	No	Yes	Yes	Yes

A novel vitreous substitute of using a foldable capsular vitreous body injected with polyvinylalcohol hydrogel	Feng, S. and Chen, H. and Liu, Y. and Huang, Z. and Sun, X. and Zhou, L. and Lu, X. and Gao, Q.	Unknown	Yes	Unknown	Unknown	No	No	No	Yes	Yes	Yes

Evaluation of a viscoelastic solution of hydroxypropyl methylcellulose as a potential vitreous substitute	Fernandez‐Vigo, J. and Refojo, M. F. and Verstraeten, T.	No	No	No	No	No	No	No	Yes	No	Yes

A new strategy to replace the natural vitreous by a novel capsular artificial vitreous body with pressure‐control valve	Gao, Q. and Mou, S. and Ge, J. and To, C. H. and Hui, Y. and Liu, A. and Wang, Z. and Long, C. and Tan, J.	No	No	No	Unknown	No	No	No	No	Yes	Yes

Effect on rabbits′ intraocular structure by cross‐linked hyaluronic formations as vitreous substitute	Gong, Yan and Chen, Kan and Wu, Yue and Guo, Xiao‐Hong and Zhang, Tao	No	No	No	No	No	No	No	No	No	Yes

An in situ‐forming polyzwitterion hydrogel: Towards vitreous substitute application	He, B. and Yang, J. and Liu, Y. and Xie, X. and Hao, H. and Xing, X. and Liu, W.	No	No	No	No	No	No	No	Unknown	Yes	Yes

Long‐Term Biocompatibility of a Highly Viscously Thiol‐Modified Cross‐Linked Hyaluronate as a Novel Vitreous Body Substitute	Hurst, J. and Rickmann, A. and Heider, N. and Hohenadl, C. and Reither, C. and Schatz, A. and Schnichels, S. and Januschowski, K. and Spitzer, M. S.	No	No	No	Yes	No	No	No	Yes	Yes	Yes

The feasibility study of an in situ marine polysaccharide‐based hydrogel as the vitreous substitute	Jiang, X. and Peng, Y. and Yang, C. and Liu, W. and Han, B.	No	No	No	No	No	No	No	No	No	No

Application of thermo‐setting gel as artificial vitreous	Katagiri, Y. and Iwasaki, T. and Ishikawa, T. and Yamakawa, N. and Suzuki, H. and Usui, M.	No	No	No	No	No	No	No	Unknown	Yes	Yes

Retinal‐detachment repair and vitreous‐like‐body reformation via a thermogelling polymer endotamponade	Liu, Z. and Liow, S. S. and Lai, S. L. and Alli‐Shaik, A. and Holder, G. E. and Parikh, B. H. and Krishnakumar, S. and Li, Z. and Tan, M. J. and Gunaratne, J. and Barathi, V. A. and Hunziker, W. and Lakshminarayanan, R. and Tan, C. W. T. and Chee, C. K. and Zhao, P. and Lingam, G. and Loh, X. J. and Su, X.	Yes	Unknown	Yes	Unknown	Yes	Yes	Yes	Yes	Yes	Unknown

Evaluation of collagen gel and hyaluronic acid as vitreous substitutes	Nakagawa, M. and Tanaka, M. and Miyata, T.	No	Unknown	Unknown	Unknown	No	Unknown	Unknown	Yes	Yes	Unknown

Super‐fast in situ formation of hydrogels based on multi‐arm functional polyethylene glycols as endotamponade substitutes	Ran, R. and Shi, W. and Gao, Y. and Wang, T. and Ren, X. and Chen, Y. and Wu, X. and Cao, J. and Zhang, M.	No	Unknown	Unknown	Unknown	No	Unknown	No	Yes	Yes	Unknown

Experimental vitreous replacement with perfluorophenanthrene	Ratiglia, R. and Berti, E. and Galimberti, D. and Bindella, A. and Schweizer, F. and Marchi, L. and Rossi, A.	No	Unknown	No	No	No	Unknown	No	Yes	Yes	Unknown

Bioinspired Fibrillary Hydrogel with Controlled Swelling Behavior: Applicability as an Artificial Vitreous	Santhanam, S. and Shui, Y. B. and Struckhoff, J. and Karakocak, B. B. and Hamilton, P. D. and Harocopos, G. J. and Ravi, N.	No	Unknown	No	No	No	Unknown	No	Yes	Yes	Unknown

Efficacy of two different thiol‐modified crosslinked hyaluronate formulations as vitreous replacement compared to silicone oil in a model of retinal detachment	Schnichels, S. and Schneider, N. and Hohenadl, C. and Hurst, J. and Schatz, A. and Januschowski, K. and Spitzer, M. S.	No	Unknown	No	No	No	Unknown	No	Yes	Yes	Unknown

The cross‐linked biopolymer hyaluronic acid as an artificial vitreous substitute	Schramm, C. and Spitzer, M. S. and Henke‐Fahle, S. and Steinmetz, G. and Januschowski, K. and Heiduschka, P. and Geis‐Gerstorfer, J. and Biedermann, T. and Bartz‐Schmidt, K. U. and Szurman, P.	No	Unknown	No	No	No	Unknown	No	Yes	Yes	Unknown

Rabbit study of an in situ forming hydrogel vitreous substitute	Swindle‐Reilly, K. E. and Shah, M. and Hamilton, P. D. and Eskin, T. A. and Kaushal, S. and Ravi, N.	No	Unknown	No	No	No	Unknown	No	Yes	Yes	Unknown

A self‐assembling peptide gel as a vitreous substitute: A rabbit study	Uesugi, K. and Sakaguchi, H. and Hayashida, Y. and Hayashi, R. and Baba, K. and Suganuma, Y. and Yokoi, H. and Tsujikawa, M. and Nishida, K.	No	Unknown	No	No	No	Unknown	No	Yes	Yes	Unknown

Antifouling Super Water Absorbent Supramolecular Polymer Hydrogel as an Artificial Vitreous Body	Wang, H. and Wu, Y. and Cui, C. and Yang, J. and Liu, W.	No	Unknown	No	No	No	Unknown	No	Yes	Yes	Unknown

Biocompatibility and retinal support of a foldable capsular vitreous body injected with saline or silicone oil implanted in rabbit eyes	Wang, P. and Gao, Q. and Jiang, Z. and Lin, J. and Liu, Y. and Chen, J. and Zhou, L. and Li, H. and Yang, Q. and Wang, T.	Yes	Unknown	No	No	No	No	No	Yes	Yes	Unknown

Feasibility study of chitosan as intravitreous tamponade material	Yang, H. and Wang, R. and Gu, Q. and Zhang, X.	No	Unknown	No	No	No	Unknown	No	Yes	Yes	Unknown

Preliminary study on retinal vascular and oxygen‐related changes after long‐term silicone oil and foldable capsular vitreous body tamponade	Yang, W. and Yuan, Y. and Zong, Y. and Huang, Z. and Mai, S. and Li, Y. and Qian, X. and Liu, Y. and Gao, Q.	Yes	Yes	Unknown	Unknown	Unknown	Unknown	Unknown	Yes	Yes	Yes

Self‐assembling hydrogel loaded with 5‐FU PLGA microspheres as a novel vitreous substitute for proliferative vitreoretinopathy	Yu, Z. and Ma, S. and Wu, M. and Cui, H. and Wu, R. and Chen, S. and Xu, C. and Lu, X. and Feng, S.	Yes	Yes	Unknown	Unknown	Unknown	Unknown	Unknown	Yes	Yes	Yes


The ROBINS‐I tool was used for human studies (*n* = 9); Table 4). The ROBINS‐I tool showed wide heterogeneity in the design of human studies. Five out of nine human studies showed “low” bias due to confounding factors. Seven out of nine human studies showed “low” bias in the selection of participants. All 9/9 studies showed “low” bias in the selection of the reporting results and “low” bias in measurement outcomes.

**Table 4:** Risk of bias assessment of included human studies using the ROBINS‐I tool.

**Study**	**Authors**	**ROBINS-I Tool**							

		**Bias due to confounding**	**Bias in selection of participants into the study**	**Bias in classification of interventions**	**Bias due to deviations from intended interventions**	**Bias due to missing data**	**Bias in measurement of outcomes**	**Bias in selection of the report result**	**Other Biases**

Perfluoro‐n‐octane as a temporary intraocular tamponade in a staged approach to manage complex retinal detachments	Barthelmes, D. and Chandra, J.	Unknown	Low	Low	Low	Low	Low	Low	Unknown

Reattachment after foldable capsular vitreous body implantation in severe retinal detachment eyes	Chen, S. and Tian, M. and Zhang, L. and Hu, C. and Liu, K. and Qin, B. and Liu, S.	Low	Low	Low	Low	Low	Low	Low	Low

Perfluorohexylethan (O62) as ocular endotamponade in complex vitreoretinal surgery	Hoerauf, H. and Roider, J. and Kobuch, K. and Laqua, H.	High	High	Low	Low	Low	Low	Low	Low

Outcomes of surgery for retinal detachment associated with proliferative vitreoretinopathy using perfluoro‐n‐octane: A multicenter study	Scott, I. U. and Flynn Jr, H. W. and Murray, T. G. and Feuer, W. J.	Low	Low	Low	Low	Low	Low	Low	Low

F6H8 as an intraoperative tool and F6H8/silicone oil as a postoperative tamponade in inferior retinal detachment with inferior PVR	Tosi, G. M. and Marigliani, D. and Bacci, T. and Romeo, N. and Balestrazzi, A. and Martone, G. and Caporossi, T.	Unknown	Low	Low	Low	Low	Low	Low	Low

Outcomes of a Foldable Capsular Vitreous Body Implantation: A Retrospective Study	Xu, X. and Ge, H. and Li, J. and Shang, W. and Ji, Y. and Yang, W. and Li, K.	Low	Low	Low	Low	Low	Low	Low	Low

Study on the effectiveness and safety of Foldable Capsular Vitreous Body implantation	Zhang, X. and Tian, X. and Zhang, B. and Guo, L. and Li, X. and Jia, Y.	Low	Moderate	Low	Low	Low	Low	Low	Unknown

Foldable Capsular Vitreous Body Implantation for Complicated Retinal Detachment Caused by Severe Ocular Trauma	Li, M. and Tang, Y. and Li, S. and Zhang, Z. and Guan, L. and Li, J. and Xu, J. and Ji, S.	Low	Low	Low	Low	Low	Low	Low	Low

Evaluation of the flexibility, efficacy, and safety of a foldable capsular vitreous body in the treatment of severe retinal detachment	Lin, X. and Ge, J. and Gao, Q. and Wang, Z. and Long, C. and He, L. and Liu, Y. and Jiang, Z.	Unknown	Low	Low	Low	Low	Low	Low	Low


#### 3.3.1. Findings

##### 3.3.1.1. Hydrogels

The biocompatibility of Healaflow, a crosslinked hyaluronic acid (HA) hydrogel, was studied in 12 pigmented rabbits (12 eyes) that underwent combined 25‐20G pars plana vitrectomy (PPV) with posterior vitreous detachment (PVD) to assess biocompatibility [7]. A total of 1 mL of Healaflow was administered. No specific disease state such as RD was studied. Rabbits were euthanized at variable time points between 42 and 105 days. Two eyes experienced iatrogenic partial RD, and two eyes developed significant cataracts from intraoperative complications. One rabbit was lost due to unrelated reasons. Postoperatively, there were no significant inflammation, infection, or corneal changes. Postoperative IOP was elevated in all rabbits (15–25 mmHg), with no reported level of significance. The iatrogenic RDs were self‐limited after long‐term follow‐up. However, the gel was only able to maintain its viscous structure in the cavity for 2 weeks before losing its viscosity during the postoperative period. Healaflow was found to have a refractive index of 1.341, and no light transmission was reported. Functional assessment on ERG revealed no significant difference in the amplitude of rod and cone responses in treated animals 3 months postinjection compared with their preoperative baseline. Hematoxylin and eosin (H&E) staining revealed normal retinal morphology. The number of apoptotic cells did not increase following hydrogel treatment as examined with TUNEL staining. Mild inflammatory reaction was seen as demonstrated by an increase in glial acidic fibrillary protein (GFAP) labeling compared with unoperated eyes.

Poly‐*N*‐vinylpyrrolidinone (NVP)‐based hydrogels were studied in 10 pigmented rabbits (19 eyes) that underwent pars plana incision with sclerostomy knife, followed by insertion of dry cylindrical specimens to assess substitute biocompatibility [8]. No PPV gauge was reported. The experiment assessed different compositions of NVP combined with either a biodegradable crosslinker (BC) or a stable crosslinker (SC, tetraethylene glycol dimethacrylate). Compositions included Material A (1:10.5 molar ratio/BC:NVP; 5 eyes), Material B (1:105/BC:NVP; 5 eyes), Material C (1:1000/BC:NVP; 5 eyes), Material D (Poly[NVP]; 1 eye), Material E (1:10.5/SC:NVP; 1 eye), Material F (1:105/SC:NVP; 1 eye), and Material G (1:1000/SC:NVP; 1 eye). The volume of the substitute administered was not reported. One rabbit that received Material A died unexpectedly 9 weeks postoperatively due to pneumonia. One rabbit (who received B and C) and two other rabbits (who received D, E, F, and G) were euthanized at 34‐ and 36‐weeks postimplantation, respectively. These rabbits′ eyes were enucleated and studied, whereas the other rabbits were not euthanized due to the authors′ plan to complete another study on substitute biocompatibility with longer follow‐up. Material A did not induce any inflammation, irritation, edema, or encapsulation postoperatively. Material B caused a cataract in one eye due to the polymeric cylinder being attached to the lens. Material C demonstrated some erythrocytes in the vitreous in one of the enucleated eyes. Implantation of poly(NVP) alone did not lead to any observed effects such as inflammation or infection. Via histopathological examination, no cellular reaction to the implants was observed. Optical outcomes and physical properties reported include the following: Material A was found to dissolve and degrade by approximately 70% within 1 week and changed from transparent to opaque white within several hours in vitro, but returned to transparent; Material B exhibited faster degradation with dissolution within 1 day; Materials C and D dissolved shortly after incubation; Material E demonstrated similar characteristics to Material A; Material F was similar to Material B; and, Material G was akin to Materials C and D. In conclusion, out of all materials, Materials A–C are the best candidates for further investigation as vitreous substitutes due to their smooth swelling and the absence of inflammation, irritation, and infection. However, these materials rapidly degraded, not making them ideal candidates for a vitreous substitute. These materials should be formulated as microspheres instead of cylinders, as was done in these in vivo studies, to avoid complications of swelling. In addition, drug release from Materials A–C is a future avenue of application due to the dampening of burst release seen with these materials.

A glycidic methacrylate‐modified HA hydrogel UV crosslinked with NVP (UV‐CHA) was studied in 12 rabbits (12 eyes) that underwent 20G PPV for RD. [9] The volume administered was not specified. The success rate of retinal attachment was found to be 67% at 6 weeks. In 33% of operated eyes, iatrogenic RD occurred during PPV. In 75% of these RDs, the retina remained attached at the final follow‐up visit. No signs of inflammation or disintegration of the hydrogels were found on slit lamp examination and fundoscopy. No other abnormal findings were reported. IOP was normal during the entire follow‐up (no significance level reported). Synthesized hydrogels were clear and had a refractive index (*n* = 1.338) that was found to be similar to human vitreous. Rheological assessment suggested adequate viscosity and elasticity for intraocular use. UV‐CHA hydrogel degraded in vitro over a period of 30 days, with a faster rate of degradation the first 15 days and then a slower rate for the next 15 days. Biocompatibility studies in vivo show that ERG B‐wave amplitudes and latencies were not significantly different between the control and UV‐CHA gel treated eyes. In vivo stability showed that the UV‐CHA gel remained fully intact after 6 weeks. In an ARPE19 cell culture treated with the UV‐CHA gel, no increase in the number of dead cells was observed with a cell viability assay. This cell biocompatibility was confirmed on a 3‐(4,5‐dimethylthiazol‐2‐yl)‐2, 5‐diphenyltetrazoliumbromide (MTT) assay and Alamar blue assay, measuring mitochondrial activity of RPE cells. Lastly, healthy retinal structure and normal cell morphology were observed on H&E for the UV‐CHA gel 6 weeks postoperatively. Notably, crosslinking can influence the biocompatibility of the gel. In this case, crosslinking HA with adipic dihydrazide (ADH) by carboxylation with *N*‐(3‐dimethylaminopropyl)‐*N* ^′^‐ethylcarbodiimide hydrochloride (EDCI) after hydrazation led to increased cell toxicity on ARPE19 cells both on cell viability and metabolic assays.

Two concentrations (24 and 40 mg/mL) of UV‐CHA hydrogel were compared with 0.9% saline as an experimental vitreous substitute. Twenty rabbits (20 eyes) underwent 20G subtotal vitrectomy, followed by injection of 1.0–1.2 mL of one of the three substances [10]. No specific disease was studied. Twelve eyes were included in the statistical analysis as five rabbits died of pneumonia, two developed iatrogenic RD, and one developed iatrogenic cataract. UV‐CHA remained stable for more than 6 weeks in the vitreous cavity of rabbits. No cataracts developed, and although IOP was increased on postoperative Week 1, it was observed to decrease gradually until it normalized to be close to the control group by postoperative Week 4 (*p* > 0.05). Throughout the observation period, all corneas remained transparent, conjunctival congestion resolved within 3 days of surgery, and the anterior chamber remained clear. The refractive index of UV‐CHA hydrogel was similar to the human vitreous body and water, although the specific value was not reported. The kinematic viscosity of UV‐CHA was 61–94 Pa·s, significantly higher than that of silicone oil (4.88 Pa·s). Schramm et al. found that high viscosity of crosslinked gel is deemed important for providing mechanical resistance to proliferative vitreoretinopathy (PVR) [9]. Despite showing no abnormalities on ocular B ultrasound compared with the control, on H&E staining, the 24 mg/mL group showed uneven rod and cone arrangements. The 40 mg/mL group showed thinning of the outer nuclear layer (ONL) with an increase in outer plexus layer (OPL) thickness. In transmission electron microscopy, the 24 mg/mL UV‐CHA treatment showed normal outer segments on rods with healthy microfilament microtubules and uniform cytoplasm, similar to the control. However, in the 40 mg/mL condition, microtubules were damaged and the ultrastructure fuzzy.

A thiol‐modified crosslinked hyaluronate (TCHA) hydrogel was studied in both soft and strong formulations as a vitreous body substitute (VBS) in 16 pigmented rabbits (16 eyes) that underwent 23G PPV followed by subretinal injection of BSS to induce RD. [11] It was compared with silicone oil injected in eight eyes with similar operative conditions and followed up for 4 weeks. The volume of substitute administered was not specified. The success rate of retinal reattachment was found to be 73% at 28 days in hydrogel treated eyes. All eyes had a moderate amount of fibrin in the anterior chamber on postoperative Day 1 (POD1). In the VBS soft group, the fibrin completely resolved in all eyes at the final follow‐up, whereas in the VBS strong group, mild amounts of fibrin were observed in 38% of animals at the end of the study. In the silicone oil group, 88% of rabbits developed a partial (one animal) or even total RD (six animals) with pronounced PVR within the first 2 weeks after surgery. In the VBS strong group, 38% of rabbits developed a new RD, 67% of which were partial RDs. No RDs were observed in the VBS soft group. The difference in RD incidence between silicone oil treated eyes and hydrogel eyes was statistically significant (*p* < 0.05). Postoperatively, IOP remained within normal limits and returned to preoperative levels by the end of the follow‐up period (no significance level reported). Cataracts were found in 29% of VBS soft, 13% of the VBS strong, and 88% of silicone oil treated eyes. The difference between silicone oil treated eyes and hydrogels was statistically significant (*p* < 0.05). Optically, VBS revealed a refractive index of 1.34 at 589 nm and 1.32 at 546 nm, respectively. Injection force, a parameter reflective of viscosity, of the hydrogel was 5.3 N for VBS soft and 14.2 N for VBS strong, respectively. This force is comparable with the force needed to inject ophthalmic viscoelastic devices during cataract surgery and lower than the reported maximum injectability threshold of 30 N [12]. In short, all physical properties were similar to normal vitreous. Postoperative OCT at 1 month demonstrated normal retinal structure and thickness for the VBS soft and strong treated eyes. ERG responses in the silicone oil treated eyes were extinct in six rabbits, reduced in one, and only unchanged in one animal, which corresponds to the extent of RD in these eyes. In contrast, the VBS strong condition had ERGs that were slightly reduced in five and strongly reduced in two animals. The VBS soft group exhibited slightly reduced ERG in three and moderately reduced ERG in three rabbits 1 month postoperatively. On histology, the VBS strong group had two animals with moderate to pronounced PVR reaction, whereas the VBS soft group had one severe case of PVR reaction and four animals with small areas of focal retinal thinning. GFAP was upregulated in both the hydrogel and silicone oil treated eyes but highest in the silicone oil condition. Lastly, Brn3a, a marker for retinal ganglion cells, was not significantly different between operated and control eyes.

In a follow‐up study, TCHA hydrogels were studied in 18 pigmented rabbits (18 eyes) that underwent 20/23G PPV with PVD to assess long‐term substitute biocompatibility [13]. The vitreous substitute was added until egress was appreciated from the second sclerotomy site. The specific volume administered was not reported. A total of 33.3% of the rabbits were euthanized at 1, 3, and 6 months, respectively. The success rate of retinal attachment was found to be 78% at 6 months. On POD1, 72% of rabbits demonstrated mild to moderate anterior chamber fibrinous reaction, which decreased to 33% and 0% of rabbits by 1 month and 3 months, respectively, after surgery. There were no signs of infection and the cornea remained clear in all rabbits throughout the duration of the study. Twenty‐eight percent of the rabbits developed iatrogenic cataracts, of which 40% occurred within 1 month after surgery, and 60% occurred between 1 and 3 months after surgery. Twenty‐two percent of the rabbits developed RD, 75% of which occurred within 1 month after surgery, and 25% occurred between 1 and 3 months after surgery. IOP stayed in the normal range throughout the entire postoperative course. However, the IOP was slightly lower on average in the experimental eye compared with the contralateral eye at 1, 3, and 6 months after surgery (at 1 month: control eye: 16.03 ± 1.8 mmHg; surgery eye: 14.05 ± 2.7; *p* = 0.22, at 3 months follow‐up: control eye: 18.95 ± 3.8 mmHg; surgery eye: 14.04 ± 2.5; *p* = 0.028), and at 6 months follow‐up: control eye: 16.74 ± 2.4; surgery eye: 13.26 ± 2.3; *p* = 0.058). On OCT, the integrity, attachment, and thickness of the retina remained the same between control and treatment conditions at all‐time points. On ERG, the a‐wave and b‐wave implicit times varied significantly between control and treated groups 1 month and 3 months postoperatively but returned to control levels at 6 months follow‐up. On H&E staining, normal retinal cytoarchitecture and morphology were observed in all 17 rabbits, except for one case with RD. No decrease in retinal ganglion cells was observed with Brn3a staining, further demonstrating biocompatibility with other retinal cell types. Despite an increase in GFAP, a marker for glial activation and fibrosis, in the treated eyes over 6 months, this could be attributed to the vitrectomy itself, which the control conditions did not receive. The refractive index was 1.338, similar to that of the human vitreous, and the transparent substitute was highly elastic, yet its viscosity allowed for easy injection and had comparable shear moduli to other substitutes. The gel was only partially present 3 months postoperation and completely degraded by 6 months.

An in situ‐formed hydrogel synthesized by crosslinking hydroxypropyl chitosan (HPCTS) with oxidized alginate dialdehyde (ADA) was studied in 10 rabbits (10 eyes) that underwent standard PPV (gauge not specified) to assess substitute biocompatibility [14]. No specific disease was studied. The volume of substitute administered was not reported. ERG analysis showed that the combined rod‐cone maximal response in the operated eyes was significantly reduced compared with those in the normal rabbit eyes, suggesting a decrease in vision (*p* < 0.05). No anterior chamber changes, vitreous hemorrhage, RD, nor choroidal detachment occurred in the operated eyes. The IOPs of the operated eyes at postoperative day 2, 4, 6, and 8 were significantly decreased (*p* < 0.05), whereas the IOPs obtained thereafter were all similar to the preoperative IOPs. The cornea and lens remained clear during the 90‐day follow‐up. Optically, the refractive index (*n* = 1.3348), light transmittance (> 80%), and density (1.015 ± 0.052 g/mL) of the hydrogel were comparable with natural human vitreous. In addition, pH (7.45 ± 0.04) and water content (98*%* ± 0.9*%*) of the gel were physiologically relevant. Rheological assessment showed that this hydrogel has high elasticity and an appropriate viscosity for vitreous substitution. Three cell lines, L929 fibroblasts, rabbit corneal endothelial cell, and rabbit RPE cell line, were used to assess cytotoxicity of the HPCTS‐ADA hydrogel. The relative growth rate of cells cultured in the hydrogel extract was similar to that of cells cultured in normal culture media as shown by MTT assays. On H&E staining, no cytoarchitectural abnormalities or inflammatory cell invasion could be detected. However, the density of photoreceptors did decrease in the operated eyes. In a follow‐up study from the same group [15], a new in situ hydrogel was made from carboxymethyl chitosan (CMCTS) cross‐linked with oxidized HA. This hydrogel was determined to be more biocompatible than the previous HPCTS‐ADA hydrogel; however, this study did not meet the sample size requirement for the number of rabbits used.

A thermo‐setting gel, WTG‐127, consisting of methylcellulose (MC) and polyethylene glycol (PEG) was studied in 10 rabbits (10 eyes) that underwent 20G PPV to assess substitute biocompatibility [16]. A total of 1 mL of substitute was administered. No specific disease was studied. The success rate of retinal attachment was found to be 100% at 28 days. However, among some of the cases excluded from analysis due to an intraoperative iatrogenic retinal tear, the injected substitute drifted under the detached retina prior to gelation. This implies the substitute may not be expected to provide an adequate tamponade effect. On postoperative Days 1 and 3, slight conjunctival hyperemia was found, but otherwise no abnormal changes occurred in the cornea, anterior chamber, lens, or posterior pole. There was no significant difference in IOP between experimental and contralateral eyes; however, no significance level was reported. At room temperature, the gel′s low viscosity facilitates handling and injection. After injection, it gels at body temperature after 50 min. Although the substance′s refractive index was not reported, its light transmissivity was 89.3% (the vitreous of human adults has been identified to have a > 90% light transmittance) [17]. The density of WTG‐127 was similar to that of the native vitreous at 1.01871 g/mL. Nevertheless, adjusting the gel′s composition can alter its viscosity, light transmission, and gelation time. On ERG, no significant differences were seen in the a‐wave and b‐wave amplitudes or latencies between control and treated eyes over the entirety of the study. On H&E staining, no histological differences were found for the gel‐treated eye 28 days postoperatively.

Three concentrations of EPC (a hydrophilic PEG, thermosensitive poly(propylene glycol) (PPG) and hydrophobic, biodegradable poly(*ε*‐caprolactone) (PCL) segments linked together via urethane bonds), 3%, 7%, and 12%, were studied in 32 New Zealand white rabbits (25 eyes) that underwent standard 23G PPV to assess substitute biocompatibility 3 to 6 months postoperatively [18]. 1.15–1.5 mL was administered. Controls included 12 operated eyes filled with BSS or four eyes filled with commercially available 20% pluronics F127 hydrogel. The success rate of retinal attachment was found to be 100% at 7 days. Postoperatively, EPC‐3% and EPC‐7% hydrogels showed no significant anterior or posterior segment adverse effects, the IOP remained normal, and the hydrogels remained optically clear in the vitreous. However, rabbits implanted with EPC‐12% hydrogel developed severe subacute inflammation by 2 weeks postsurgery associated with elevated IOP. 75% of EPC‐12% rabbits had raised IOP of ≥ 30 mmHg at postoperative Day 14. In 67% of these rabbits, IOP normalized (< 30 mmHg) by postoperative Day 30. Optically, transparency at body temperature and refractive index of the hydrogel were similar to normal human vitreous, which is possibly due to its > 90% water content (*i* = 1.339–1.344). The viscosity of the hydrogel depends on the EPC concentration, with a sol‐gel transition state for EPC‐3% and EPC‐7% at 25°C, and at 4°C for EPC‐12%. All gels rapidly formed a gel in the vitreous cavity at 37°C, which was essential to its handling and clinical function. EPC‐7% showed better in vivo functional results than EPC‐3%, which had notable scotopic and photopic ERG abnormalities with inner retinal function loss that had minimal recovery 3 months after surgery. EPC‐7% showed only mild loss of scotopic b‐wave and photopic 30 Hz flicker response amplitudes at 1 month with complete recovery by 3 months. To validate EPC‐7%′s potential as retinal tamponade, RD was surgically induced in 2 nonhuman primates, following which 1–1.5 mL of EPC‐7% was injected into the vitreous cavity. No anterior segment inflammation or cataracts were observed and OCT imaging revealed normal retinal thickness and architecture. IOP remained within a normal range (12–17 mmHg) and the retina remained attached for 12 months without prone positioning. One month postsurgery, mild cone dysfunction was observed on full‐field ERG, which fully recovered by 12 months. Lastly, histological analysis on H&E stained tissue demonstrated normal retinal cytoarchitecture in EPC‐7%. In a follow‐up study from the same group [19], it was determined that the molecular weight (MW) of the polymers used to make EPC affected the properties of the hydrogel. Low MW polymers resulted in retinal atrophy in a rabbit model, whereas high MW polymers caused the hydrogel to turn opaque. In another follow‐up study [20], glycerol was added to the EPC hydrogel formulations to create hyperbranched EPCG hydrogels. Low‐branched hydrogels were determined to be biocompatible in a rabbit model for 4 months, but high‐branched hydrogels created vitreal haziness and caused retinal damage.

Four concentrations of PEG hydrogel (8, 9, 10, and 20 mg/mL) were studied in 34 chinchilla rabbits (34 eyes) that underwent 25G PPV to assess substitute biocompatibility [21]. A range of 0.8–1.2 mL was administered. The success rate was found to be 100% at 6 months. Mild anterior segment inflammation was seen at 1 and 7 days after surgery in the 9 mg/mL and 10 mg/mL groups, which was comparable with the normal control. The 20 mg/mL group had an inflammatory reaction in the anterior segment, most pronounced on the first postoperative day, which resolved by postoperative Day 7. A decrease in IOP was seen in the first 3 days after surgery in all operated groups. Otherwise, there were no abnormal findings of the cornea, conjunctiva, or lens throughout the evaluation period. The average IOP value of 30 mmHg surpassed the normal range in the 20 mg/mL group at postoperative Month 3. Optically, light transmittance was above 90% for all concentrations at 37.1°C and the refractive index was 1.3364–1.3390, both of which are comparable with humans. The 9 mg/ml gel had the best biocompatibility when injected into the subconjunctival tissue with minimal degradation at 3 weeks postoperatively. In a RD model in rabbits, the 9 mg/mL gel was injected and evaluated at 6 months after surgery. Fundus fluorescein angiography (FFA), OCT, and B‐scan ultrasonography demonstrated the gel′s ability to heal retinal breaks and clear retinal photocoagulation. ERG measurements and H&E staining were also normal at 6 months postoperation for the 9 mg/mL gel treatment compared with the control. This is in contrast to the 20 mg/mL gel condition that showed both reduction in ERG amplitudes 3 months postoperatively and chronic inflammation and degeneration of the optic nerve fiber layer on H&E staining 1 month after surgery.

A thiol‐containing hydrogel, in 2% (*n* = 9) and 3% (*n* = 1) concentrations, used a disulfide cross‐linker that was then reduced to produce an injectable thiol‐containing polymer solution was studied in 10 black rabbits (10 eyes) that underwent 25G PPV to assess substitute biocompatibility and compared with an air‐filled control (*n* = 4) [22]. The amount of substitute administered was not reported. The success rate of retinal attachment was found to be 90% at 7 days due to one case of iatrogenic RD. Trace cells were found in all operated eyes on POD1, which was resolved by POD7. On POD1 and 7, no significant anterior or posterior inflammation and no elevated IOP on palpation were found. There was no evidence of recurrent RD nor SRF. Optical outcome reported a refractive index of 1.33. The hydrogel had viscoelastic properties akin to natural vitreous. Fundus photographs of the macula and periphery with the vitreous substitute showed no evidence of retinitis, vasculitis or pigmentary changes postoperative Day 7. ERG responses were also normal in the hydrogel injected eyes in comparison with the contralateral control. This was similar to the ERG responses in the air endotamponade group versus their respective contralateral eye. On H&E histology, the retinal structures are preserved with no vacuolization.

Two compositions of a two‐component hydrogel composed of a thiolated fibrillary gellan and a semiflexible HA (CoP), 0.9G_12CoP and 1.5G_10CoP hydrogel, were studied in 22 rabbits (22 eyes) that underwent 23G PPV to assess substitute biocompatibility [23]. The thiolated gellan and the CoP were separately warmed at 48°C for 15 min to remain a liquid for injection (sol−gel transition temperature: 40°C). A range of 0.5 − 0.6 mL of the substitute was injected through an infusion line into a partially empty rabbit vitreous cavity. The temperature of the polymer solution decreased to∼42°C as it traveled through the infusion line. The success rate of retinal attachment was found to be 100% at 30 days. No specific disease was studied. Conjunctival injection was seen in all groups as of POD1, and this was resolved by 1 week after the surgery. Within the first month, conjunctival injection was observed with no significant anterior segment inflammation found. Anterior segment remained normal with IOP within normal limits. At 30 days postoperation, 27% of 0.9G 12CoP‐treated, and 55% of 1.5G 10CoP‐treated rabbits showed signs of slight posterior polar cataract. These were posited to be iatrogenic. One rabbit treated with 1.5G 10CoP had vitreous hemorrhage at postoperative Day 30, but this occurred at the time of surgery. Otherwise, the posterior exam of all experimental cases was normal. Preoperative and postoperative IOPs were comparable for all experimental groups. The 0.9G 12CoP group had a significant decrease in IOP on POD1 (significance level not reported), but this was resolved by postoperative Day 4. The immediate postoperative IOPs for all experimental groups were slightly higher than reported rabbit IOPs in the literature, and the increased IOP subsided by POD1. The gels were transparent viscoelastic solids with optical and physical characteristics similar to the native vitreous. The refractive indices, light transmissions, and viscosities of the compositions were not reported. The reported stability of the hydrogel is 30 days. The normalized degree of swelling was 1.6 for the two‐component hydrogel, 0.6 higher than its nonswelling thiolated gellan component and 0.4 degrees lower than the highly swelling CoP component. On OCT, no significant difference was seen between the retinal thickness of the hydrogel treated eye, unoperated eye, and silicone oil control. ERG responses of the experimental and control groups were not significantly different. Therefore, the photoreceptor function of the hydrogel treated, silicone oil injected, and unoperated control eyes was normal. On H&E histological examination, the integrity of the retinal cytoarchitecture remained intact without any retinal edema, atrophic changes or hemorrhage. These pathological changes were given the quantitative score of 1 (0 = no pathological change, 4 = severe pathological change). Minimal mononuclear cell inflammation was seen in the vitreous, which could have resulted from the focal trauma to the posterior lens capsule or the suture line during surgery. In a follow‐up study from the same group [24], biocompatibility was demonstrated in a rabbit model for up to 4 months; however, this study did not meet the sample size requirement for the number of rabbits used.

A self‐assembling peptide hydrogel, PanaceaGel SPG‐178, was studied in 15 rabbits (15 eyes) that underwent 25G PPV to assess substitute biocompatibility and compared with BSS controls (*n* = 6) [25]. The volume administered was not specified. The success rate was found to be 100% at postoperative Day 3 (*n* = 15), POD7 (*n* = 11), POD28 (*n* = 7), POD84 (*n* = 5). On POD 1, 3, 7, 14, 21, 28 (1 month), 56 (2 months), and 84 (3 months) no infection, cataract, or significantly different IOP was noted (*p* > 0.05). The refractive index of the vitreous substitute was 1.3339 and the visible light transmission rate was 96.7%. The substitute had a greater storage modulus (G^′^) than the loss modulus (G^″^) at all frequencies, indicating a gel‐like behavior. Examination on slit‐lamp and fundoscopy demonstrated no hemorrhage, inflammation, or vitreous opacities in the gel treated eyes. The a‐ and b‐wave amplitudes and implicit times were not significantly different between control and vitreous substitute groups. Lastly, H&E staining revealed no inflammation, degeneration, or structural changes in the retinal architecture in control and gel treated eyes 3 months postoperatively. It is unknown if PanaceaGel SPG‐178 is biodegradable.

Zwitterionic poly(*N*‐acroyl glycinamide) (PNAGA) and poly(*N*‐acroyl glycinamide‐co‐carboxybetaine acrylamide) (PNAGA‐PCBAA) supramolecular hydrogels prepared through photoinitiated copolymerization were studied in 10 rabbits (10 eyes, five per group) that underwent 22G PPV to assess substitute biocompatibility [26]. A total of 0.5–1 mL was administered. No specific disease was studied. The success rate was 100% at 16 weeks. In the PNAGA‐PCBAA‐10‐4 hydrogel group, no vitritis, uveitis, retinitis, endophthalmitis, vitreous hemorrhage, or RD was observed. However, there were no unambiguous vessels and tissue structures that could be appreciated for PNAGA hydrogels at postoperative Weeks 8 and 16. Potential explanations include intraocular inflammatory response, vitreous hemorrhage, retina detachment, or low transmittance of PNAGA hydrogel. No changes were found in the cornea or ciliary bodies. The PNAGA hydrogel elevated IOP, with no reported significance level. PNAGA had a refractive index similar to natural vitreous (*n* = 1.3572), but a low 53.3% and 51.1% light transmission rate preoperatively and 6 months postoperatively, respectively. Conversely, the light transmittance of PNAGA‐PCBAA hydrogels at 6 months postoperatively was 92.9%. In terms of viscoelastic properties, G^′^ was greater than G^′′^ at all frequencies from 0.01–10 Hz, with no significant crossover, suggesting a “gel‐like” behavior of the hydrogels. Subcutaneously implanted PNAGA hydrogel into the C57BL/6 mice demonstrated significant inflammatory cells 1 month postoperatively, whereas subcutaneous PNAGA‐PCBAA‐10‐4 showed fewer inflammatory cells. PNAGA‐PCBAA‐10‐4 also performed better in fibrotic response. Both formulations have minimal swelling, with constant volumes in PBS. B‐scan ultrasound demonstrated normal retinal integrity for all conditions, except the PNAGA hydrogel group, which exhibited echoes from a RD or intraocular foreign body reaction. Similarly, ERG responses were comparable between the control and PNAGA‐PCBAA‐10‐4 group, whereas in the PNAGA hydrogel‐implanted eyes, the b‐wave amplitudes were significantly decreased in scotopic and photopic ERG. H&E staining revealed healthy structure and cell morphology of the cornea, ciliary body, and retina in the normal, sham‐operated and PNAGA‐PCBAA‐10‐4 groups 16 weeks after surgery. However, the PNAGA hydrogel group demonstrated a defective and dissociated retina with a fuzzy corneal structure. In a follow‐up study from the same group [17], a PCB‐OAA zwitterionic hydrogel was determined to be biocompatible in a rabbit model for 6 months; however, this study did not meet the sample size requirement for the number of rabbits used. In addition, the hydrogel was not biodegradable.

A 3% polyvinyl alcohol (PVA)/chitosan hydrogel with or without encapsulation of 5‐fluorouracil (5‐FU) within poly(lactic‐co‐glycolic acid) (PLGA) microspheres was studied in 12 rabbits (12 eyes) that underwent 25G PPV to assess substitute biocompatibility [27]. Approximately 0.9 mL was administered. No specific disease was studied. The success rate was not reported. No complications, such as corneal opacity, keratopathy, or posterior synechiae, were observed within 24 weeks. The experimental groups had a significantly increased IOP at post op Weeks 1 and 4, which decreased IOP by 4 months (*p* < 0.05). The refractive index of the substitute ranged from 1.33–1.62, with a light transmittance from 83%–93%, and a density from 1.0142–1.1247. At all frequencies, for all PVA/chitosan hydrogel concentrations,G^′^ was higher than G^″^, and the preparation showed gelatinous characteristics. The curves of G^′^ and G^″^ were almost parallel, and there was no intersection. Five and 7% PVA/chitosan hydrogels were determined to be unsuitable as a substitute because their rheological parameters were higher than the average steady state moduli of G^′^ = 2.8 ± 0.9 Pa and G^″^ = 0.7 ± 0.4 Pa (1 Hz) of the porcine vitreous. In vivo, 3% PVA/chitosan hydrogel and 3% PVA/chitosan hydrogel plus 5‐FU PLGA microspheres (for PVR treatment) were tested. The 3% PVA/chitosan hydrogel loaded with 5‐FU PLGA microspheres performed better than both the 3% PVA/chitosan and control groups, as demonstrated on B‐scan ultrasound and fundoscopic examination. In addition, although epiretinal membrane thickening and RPE cell proliferation were observed in the 3% PVA/chitosan and control groups, there were no histological abnormalities for the group loaded with 5‐FU PLGA microspheres. Therefore, loading 5‐FU PLGA microspheres into the 3% PVA/chitosan hydrogel has potential in reducing complications in patients with PVR. Important to note is the presence of water‐like substances in the vitreous cavity at the end of the study, indicating some degree of hydrogel hydrolysis.

Vitargus ABV‐1701, a hydrogel made of oxidized HA crosslinked with adipic acid dihydrazide (ADH), entered a Phase I clinical trial and was studied in 11 human patients: three patients had RD, seven patients had vitreous hemorrhage, and one patient had both [28]. All patients required vitrectomy and had a best corrected visual acuity (BCVA) of 20/40 to 20/2000 before surgery. Following vitrectomy, the hydrogel was injected as a liquid, and gelled in situ. The mean BCVA of the patients improved after surgery by 31.9 ± 32.8 letters at Day 1, 21.4 ± 44.0 at Day 7, and 31.9 ± 32.8 at 1 month (*p* < 0.05), compared with a baseline of 16.5 ± 21.2. Three in patients who experienced elevated IOP in the days following surgery and required medical interventions. No toxicity to ocular tissues was observed. The hydrogel has a refractive index of 1.34. A Phase 2 clinical to demonstrate safety and efficacy in uncomplicated RD patients is to be conducted in both Australia and Thailand and will enroll at least 40 patients.

A novel HA‐PEG oxime‐crosslinked hydrogel was studied in a total of 40 rabbits (40 eyes) who underwent 23G PPV [29]. 1 mL of the hydrogel was injected into the vitreous cavity. Over a 56‐day period, the rabbit retina remained healthy and intact, with no detachments. For all rabbits, mean IOPs were reported within normal range (13.5–25.92 mmHg). The density (1.01 g/mL), refractive index (1.356), and transparency of the hydrogel were comparable with that of the native vitreous. In addition, this hydrogel was minimally swelling over 28 d and injectable by hand through a 23G needle for 10 min. Notably, this gelation time could be tuned by varying the concentration of functional groups on the HA polymer. The surface tension was also significantly higher than that of silicone oil, indicating that the hydrogel will not migrate into retinal tears or the anterior chamber. Photoreceptor cells isolated from Nrl knockout mice and treated with the HA‐oxime hydrogel for 24 h did not show an increase in dead cells with a live/dead assay. In an explant study, flat‐mounted mouse retinas treated with the HA‐oxime gel for 24 h were dissociated and assessed for viability on flow cytometry, which showed no increase in cell necrosis or apoptosis. Lastly, human embryonic stem‐cell derived RPE demonstrated 96% viability after 24 h incubation with the HA‐oxime gel. The number of nuclei in the retinal layers including the ganglion cell layer and the inner and ONLs remained similar among all timepoints, demonstrating the in vivo biocompatibility of the HA‐oxime hydrogel. Retinal function also remained intact in the treated rabbits as indicated by a normal index of retinal sensitivity and healthy ERG responses. Lastly, healthy retinal layers were shown on OCT in the hydrogel‐treated animals. The authors suggest that the observation of normal IOP in these rabbits is due to the slow hydrolysis of oxime linkages as opposed to the faster hydrolysis of hydrazones in Vitargus.

Recent experimental studies in Table 2 explored three additional hydrogels evaluated in rabbit eyes (*n* < 10 per study). These included a transparent alginate–PVA in situ self‐healing gel achieving 75% retinal reattachment versus 57% with silicone oil controls [30] a self‐crosslinking HA hydrogel maintaining retinal attachment and normal ERG amplitudes with mild, self‐resolving anterior inflammation [31], and an antioxidant poly(ethylene glycol) diacrylate (PEGDA) hydrogel releasing ascorbic acid and glutathione that preserved retinal architecture and IOP with > 90% light transmittance [32]. These studies did not meet inclusion criteria but are summarized in Table 2 to highlight the most recent vitreous‐mimetic materials.

##### 3.3.1.2. Polymers

E10KDC18, a PEG compound end‐capped with an octadecyl group, was studied in 10 rabbits (10 eyes) who underwent a 21G PPV to treat retinal tears and as an effective vitreous substitute. The amount administered was not reported. E10KDC18 was a successful tamponade in 100% of the induced retinal breaks; the follow‐up date was not reported. The polymer was chemically inert before and after injection into the vitreous and exhibited sufficient rigidity to tamponade the retina. The transmission of light was similar to that of the native vitreous (> 90%) with a refractive index of 1.353, which is also similar to the native vitreous (1.336). Percentage viability of murine catecholaminergic cells after 48 h incubation with E10KDC18 is close to 100% with no morphological changes. IOP remained similar to the saline‐injected eyes throughout the 3‐month observation period. Histopathologic examination demonstrated no disorganization of the retinal layers, no loss of nerve fibers or infiltration of inflammatory cells in the polymer injected eyes.

Hydroxypropyl methylcellulose (HPMC), an inert polymer, was evaluated as a vitreous substitute in 16 rabbits (16 eyes) in the management of complicated RDs [33]. A gas 30G vitrectomy with C_3_F_8_ was performed. A total of 0.5 mL was administered. The success rate was not reported. HPMC treated retinal pigment epithelial cells showed persistent cell density and confluency on Day 28 when compared with controls. When HPMC was injected into the eye, the viscosity dropped nearly one half its room temperature value when it reached body temperature. The HPMC solution was also diluted by intraocular fluids and found to not be able to seal retinal holes. At 6 weeks, 50% of rabbits had a 180° RD. The study showed that it is unlikely that HPMC solution will be useful as a long‐term vitreous replacement due to its rapid intraocular decrease in viscosity. Optical outcomes reported were not reported.

Fluorescein isothiocynate (FITC)‐labeled collagen and fluorescein‐labeled hyaluronic acid (FL‐HA), or a mixture thereof, was injected into 24 rabbit eyes (*n* = 8 per group) after a PPV using an Ocutome and a fiberoptic endoilluminator in a prospective study studying complicated RDs [34]. A total of 0.5 mL was administered. The success rate of the tamponade was not reported. Upon injection of FITC‐collagen, collagen accumulated in the vitreous and was observed to be in direct contact with the internal limiting membrane. One month after injection, light microscopy showed no marked retinal abnormalities. No marked IOP increase was observed at 7, 14, 30 and 60 days postoperation. The results indicated that a mix of collagen and HA is safe and effective for 3 months as a vitreous substitute in rabbits. The FL‐HA demonstrated the shortest half‐life (2.09 d), followed by FITC‐collagen (5.70 d), and lastly the mixed gel (8.41 d). The rapid clearance of the polymers, due to their uncrosslinked nature, limits their potential use as a vitreous substitute. Optical outcomes demonstrated that FITC‐collagen maintained its transparency during the 3‐month period. No refractive index or light transmission was reported. ERG measurements showed normal a‐ and b‐wave amplitudes and latencies for vitreous substitute conditions, although follow‐ups were only done until 7 days postoperation. On histology, none of the three polymer conditions exhibited abnormal retinal cytoarchitecture 3 months after injection.

Chitosan solution was investigated in a prospective study in 12 chinchilla rabbits (12 eyes) receiving a subtotal vitrectomy. Between 1.2–1.5 mL was administered [35]. At 30 days, 100% of chinchilla rabbits had anatomical attachment of the retina without proliferation. Chitosan was found to demonstrate mild conjunctival congestion and mild anterior uveitis with an increase in IL‐6, IL‐8, nitric oxide at both 7 days and 30 days postoperation, but this difference was not significant compared with the control group. No morphologic changes were seen in either group under light microscopy in the cornea, ciliary body, and lens. The outer plexiform layer of the retina thinned, but no significant degeneration, necrosis, karyopyknosis, or lysis were found via ultrastructural microscopy. There was also no significant difference in IOP at different time points between the experimental and control group (*p* > 0.05). Optical outcomes reported that the cornea, lens, and vitreous cavity remained transparent throughout the entire study. It is reported that the half‐life of the chitosan solution is 21 days, although the authors do not specify if that half‐life is in vitro or in vivo.

##### 3.3.1.3. Liquids

Perfluoro‐n‐octane (PFO) was studied as a short‐term postoperative intraocular tamponade in management of 17 complicated RDs (17 eyes) in humans who underwent 23G PPV [36]. The amount administered was not reported. The success rate was found to be 94% at 14 days. 16/17 eyes (94%) had complete reattachment. All eyes retained or improved visual acuity. Complications reported included 2/17 eyes (11.8%) with long‐term low IOP of 5 mmHg. Optical outcomes were not reported.

PFO was studied in a prospective interventional multicenter study of 555 patients to evaluate the management of RD associated with PVR with 30G PPV [37]. The amount administered was not reported. The success rate was found to be 77% at the final one‐year follow‐up. Among the 465 eyes with final visual acuities available, 274 (60%) eyes had improved VA, 106 (23%) remained stable, and 85 (18%) worsened. The retina was attached in 279 (78%) eyes, and retained PFO was noted in 20 (6%). Two hundred thirty‐eight of 555 (43%) eyes had to undergo reoperation for the RD, and at 6 months, corneal edema, elevated IOP, and hypotony were noted in 26 of 356 (7%), 6 of 356 (2%), and 48 of 356 (15%) eyes, respectively. Optical outcomes reported were not reported.

Perfluorophenanthrene (PFP) was examined as a short‐, medium‐, and long‐term tamponade vsBSS [38]. 24 rabbit eyes were evaluated after undergoing a 21G PPV for the management of complicated RDs. A range of of 0.8–1.0 mL was administered. The success rate was not reported. Histological examination showed progressive damage of the chorioretinal tissue at POD7. At POD60, there were marked changes demonstrated to the retinal architecture. In terms of clinical outcomes, posterior capsular cataract developed in 4/24 (16.7%); RD was found in 6/24 (25%) of the eyes were PFP had been removed from the vitreous after 1 week, but no changes in IOP were present in any of the eyes. Optical outcomes reported were not reported.

Perfluorohexyloctane (F6H8) was evaluated in a retrospective review of 22 patients (22 eyes) who underwent PPV with the Millennium vitrectomy system (either 23 or 25G) for postoperative tamponade for interior RDs with interior PVR [39]. Different ratios of F6H8 to silicone oil were tested. The amount administered was not reported. At mean follow‐up of 22.63 months, F6H8 was successful in flattening the retina in all cases and 21/22 patients (95.45%) achieved a complete retinal reattachment. However, recurrent inferior PVR was observed in 2 (ratios of 70/30 and 60/40, respectively) and recurrent tractional RD with superior PVR occurred in three patients (30/70 ratio). The optimal ratio of F6H8/silicone oil was found to be between 50/50 and 30/70. F_6_H_8_ has a density of 1.3 g/mL and is a transparent mixture present with all ratios besides 70/30 and 60/40.

Perfluorohexylethan (O_62_), a partially fluorinated alkane, was investigated in a prospective clinical study of 11 patients (11 eyes) in the study of inferior pathologic conditions, PVR, RRD with inferior tears and inferior giant tears who underwent 20G PPV [40]. The amount administered was not reported. At an average of 16 months, O_62_ was able to flatten and reposition the central retina initially in 100% of eyes. However, differences in physical properties led to difficulties in reattaching the peripheral retina in 45.5% of eyes. Recurrent RDs with PVR developed in four of 11 eyes under the tamponade (two superiorly, one inferiorly, and one total). Due to its early emulsification and reported uveitis reactions, O_62_ was deemed unsuitable as a long‐term ocular endotamponade. Physical properties reported were a refractive index of 1.29 and a viscosity of 0.75 mPa, similar to that of well‐known PFCLs.

Medium‐chain triglycerides (MCTs) were studied in 28 rabbits (28 eyes) that underwent 20G PPV for PVD [41]. A total of 0.8–1.5 mL was administered. The success rate of retinal attachment was 71% at 90 days, excluding intraoperative iatrogenic RDs. No conjunctival, external eye, or anterior chamber abnormalities were seen. Seven percent of eyes developed a Grade 2 inflammatory reaction in the vitreous, which was improved but not completely resolved 2 weeks postoperatively. Forty‐one percent of eyes exhibited signs of a mild inflammatory reaction in the inferior vitreous, which was most pronounced at POD7, and gradually decreased thereafter. During the vitrectomy, inferior RDs occurred in eight eyes, the substitute was misinjected into the anterior chamber of one eye, and the vitreous cutter touched the lens during vitrectomy in two eyes leading to cataract development. Otherwise, there was no cataract development in any of the eyes in the postoperative period. IOP remained normal in all rabbits during the entire follow‐up (no significance level reported). In all of the aforementioned iatrogenic RDs, the superior retina remained attached with no observed passage of MCTs under the retina. The substitute remained optically clear with retained viscosity during the surgery and throughout the postoperative period. The density of the triglycerides at 20°C was 0.94–0.95 g/mL for a viscosity at 20°C of 27–33 mPa, and the refractive index was 1.449–1.451. In a follow‐up study from the same group [42], MCTs were used in a minipig RD model. Ten minipigs (10 eyes) underwent vitrectomy followed by RRD, retinal flattening, retinopexy, and MCT injection, whereas five minipigs (five eyes) underwent vitrectomy followed directly by MCT injection. For the minipigs that received RRD, after 45 d the retina was successfully reattached in seven eyes, whereas redetachment occurred in two eyes and one eye could not be assessed. Histology showed no signs of retinal toxicity. ERG showed no significant difference between eyes that received MCT and control eyes.

#### 3.3.2. Devices

A FCVB is a modified polysiloxane elastomer that can be injected with various solutions to fill the potential space following 23G PPV as a vitreous substitute. This device has been studied in RD, PVR, and ocular trauma in rabbit and human models.

In 20 rabbits (20 eyes), FCVB filled with either BSS or silicone oil was investigated as PVR treatment [43]. The success rate was 100% for the FCVB eyes, whereas in the silicone oil group 50% (5/10) of RDs reoccurred. The implantation of the FCVB did not require a routine fluid‐air exchange. No complications such as retinal redetachment, vitreous hemorrhage, or intraocular inflammation took place in the FCVB group. A drawback of FCVB is its refractive index, which would result in emmetropia clinically [43]. Similarly, this group investigated whether PEG used in the FCVB (versus BSS in the control eye) could serve as a long‐term vitreous substitute in 30 rabbits (12 eyes received FCVB with PEG, nine eyes PEG alone, and nine eyes BSS) with RDs who underwent 23G PPV [44]. In all FCVB eyes, the FCVB remained in contact with the retina over 3 months, with no documented ocular cytotoxicity. However, there was a higher incidence of cataracts and lens subluxation. Twenty‐five percent of FCVB‐implanted eyes developed cataracts, versus 11.1% in both the PEG and BSS‐implanted groups during the entire observation period. Clinical and pathologic findings showed that FCVB filled with PEG had good biocompatibility in rabbit eyes with no elevation in IOP, inflammation, vitreous hemorrhage, and redetachments as compared with the control group. Optically, the transparency was > 93% and absorbance values were not significantly different than the control BSS group [44].

Another prospective study in 18 rabbit eyes evaluated FCVB injected with either saline or silicone oil as potential PVR treatment. The success rate was found to be 100% in all FCVB eyes at POD90 [45]. On histological examination, no evidence of pathologic changes or structural abnormalities in the eye was found. No inflammation or hemorrhage occurred; however, cataracts did form in all conditions (50% in FCVB + saline group, 83% in FCVB + silicone oil, and 40% in silicone oil alone conditions). A prospective study was performed to determine whether the FCVB had long‐term impacts on retinal vasculature. A 20G PPV was performed in 46 rabbits, out of which 23 eyes were injected with FCVB and silicone oil combination, and 23 with a silicone oil tamponade alone. After 180‐day follow‐up, there were no significant differences in HIF‐1*α* and VEGF levels between the control and experimental groups [46]. All eyes showed clarity in the vitreous cavities, although no other optical outcomes were reported [47].

In humans, the FCVB filled with BSS was studied in 11 patients (11 eyes) who underwent a 23G PPV for RDs [47]. At 3 months, retinal reattachment rate was 73%. Retinal reattachments were observed in 8–11 eyes after FCVB removal. Over the 3‐month period, visual acuity did not show a significant difference [47]. No serious complications occurred, but two cases anterior chamber hyphema was observed. Following the FCVB was studied in 27 patient eyes who underwent a 23G PPV for RDs. The retinal reattachment rates with the FCVBs reached nearly 93%. At the 6‐month follow‐up, preoperative (1.30 ± 1.20) and postoperative (0.63 ± 0.79) vision was significantly improved (*t* = 3.03, *p* = 0.005). Complications include the FCVB breaking during surgery (*n* = 1) and drainage tube exposure (*n* = 1) but no other complications such as inflammation, elevated IOP, endophthalmitis, sympathetic ophthalmia and rejection of the FCVB were detected [48]. For complicated RD caused by severe ocular trauma. FCVB was studied in 28 patients following 23G PPV. In 100% of cases, the FCVB was successfully implanted, and the retina was reattached. The postoperative BVCA improved in 7/28 of the cases and remained unchanged in the other 21/28. For complications, there was a statistically significant difference in IOP before and after surgery, 7.01 ± 2.43 and 8.54 ± 2.93 mmHg, respectively. Postoperative patient satisfaction was also significantly better than preoperative satisfaction [49]. A follow‐up study of 18 of these patients (18 eyes) at 12 months found the success rate of reattachment to be 100%. VA improved in 7/18 of the eyes (39%), stayed the same in 10/18 eyes (56%) and decreased in 1/18 eyes (6%). No elevated IOP, inflammation, rejection, displacement, or rupture were observed. 8/18 eyes preserved a clear cornea with follow‐up [50]. Another retrospective analysis in 20 patients who underwent PPV and FCVB implantation. All patients had a history of severe ocular trauma or silicone oil dependent eyes. At 12 months, 30% (6/20) of the treated eyes achieved retinal reattachment. No significant differences were noted in visual acuity, and 50% of the eyes maintained normal levels of IOP after surgery. Although no severe complications were associated with the FCVB, some patients suffered mild hemorrhage and omental proliferation caused by previous ocular damage [51]. Optical outcomes were not reported.

Lin et al. studied a FCVB injected with silicone oil for severe RD that could not be easily reattached with silicone oil tamponade alone [47]. Over 1 year, the silicone oil‐filled FCVB was shown to be effective and safe in three eyes as a vitreous substitute, avoiding complications induced by silicone oil, such as glaucoma, corneal degeneration, and silicone oil emulsification during a 12‐month implantation time. There was a slight IOP elevation after FCVB implantation compared with those of preoperative eyes. The fundus examination and OCT showed that the FCVB was well distributed in the vitreous cavity and evenly supported the retina. Retinal reattachment was successful in all three eyes at the 12‐month examination. No significant decrease in the density of corneal endothelial cells from baseline was found. UBM showed that the FCVB smoothly contacted but did not crush the ciliary body. At 3 years follow‐up, authors found that the silicone oil‐filled FCVB was effective and safe as a vitreous substitute over a 3‐year observation period. At the 3‐year follow‐up, fundus photography and OCT showed retinal reattachment in all three cases, with stable IOP. There were mild improvements in visual acuity from baseline. OCT revealed decreased retinal thickness and an altered retinal structure in the implanted eyes compared with the control eyes. No keratopathy, glaucoma, silicone oil leakage, or silicone oil emulsification occurred during the observation period.

In addition, studies have compared 3% PVA hydrogel versus FCVB filled with 3% PVA (FCVB + PVA) versus BSS in 18 albino rabbits (18 eyes, six per group) that had 20G PPV to assess substitute biocompatibility [52]. A total of 1.1 mL of 3% PVA or BSS was injected into the vitreous cavity, and 1.4 mL of 3% PVA was injected into the FCVB in the FCVB + PVA groups. No specific disease was studied. After 180 days of long‐term tamponade, the success rate was 100% in all groups. Postoperatively, anterior chamber inflammation was noted in all groups, but most intense in the FCVB + PVA group, which recovered within a week with treatment. There was increased cataract development in the PVA hydrogel group (50% vs. 17%) compared with the BSS group, and a significant decrease in IOP after 180 days in the PVA only group [52]. Postoperatively at 7, 14, 30, 60, and 90 days, no evidence of uveitis, vitritis, retinitis, endophthalmitis, vitreous hemorrhage, or RD was noted in any groups. Although the vitreous was clear in all groups at postoperative day 90, the vitreous cavities appeared relatively blurry in 50% of the PVA hydrogel group due to the abovementioned cataract formation. Of note, lensectomy was performed in all PVA + FCVB cases during the PPV as the authors′ prior study demonstrated severe cataract development frequently in eyes that receive FCVB. Optical outcomes reported were a refractive index of 1.3361 and light transmission of 93% for 3% PVA. In terms of physical properties, there was appreciable degradation of the substitute in the PVA group and a decrease of viscoelastic properties after 180 days of tamponade in vivo. Conversely, in the PVA + FCVB group, the PVA retained its rheological features without degradation at POD180.

## 4. Discussion

In current clinical use, silicone oils, SFAs and gasses are used for endotamponades for promoting retinal reattachment during the treatment of vitreoretinal pathologies. However, they deviate in their similarity to the native vitreous in their refractive index, density, and hydrophilicity leading to posoperative consequences including raised IOP, inflammation, lack of visual sharpness, and cataract formation. Thus, ideal vitreous substitutes are those which are similar to the native vitreous in both function and structure (biocompatible, optically transparent, and maintain normal IOP) and also lack negative characteristics such as biodegradation. To our knowledge, this is the first systematic review examining the optical, biological and clinical parameters of experimental vitreous substitutes in animal and human models using literature from 1990 to 2025. We report on novel experimental vitreous substitutes including hydrogels, polymers, liquids and devices which show promise for additional large‐scale investigation in animal or human experiments.

In principle, hydrogel‐based vitreous have favorable properties such as their transparency and biocompatibility, and decreased risk of postoperative complications such as cataract formation. The review described positive biologic, physical, and chemical properties of Healaflow crosslinked HA, PVP, WTG‐127 MC thermogel, fibrillary gellan semiflexible polyelectrolyte, UV‐crosslinked HA, thiolated crosslinked HA, HPCTS/ADA, PEG/PPG/PCL, PEG, PanaceaGel SPG‐178 self‐assembling peptide, UV‐crosslinked GMHA, thiolated co‐PAA, PNAGA, and PNAGA‐PCBAAA. Several recent rabbit studies summarized in Table 2 report promising optical and biocompatibility profiles for alginate–PVA, self‐crosslinking HA, antioxidant PEG, and dual‐crosslinked betaine hydrogels, although the small sample size limits the strength of the conclusions. Although many were able to effectively tamponade the retina, several of the HA‐based vitreous substitutes were demonstrated to best act as short‐term substitutes due to their loss of viscosity and enzymatic biodegradation in the eye. Positive characteristics for a vitreous substitute were also described in additional polymers, including E10KDC18 octadecyl‐capped PEG, HPMC, FITC‐collagen, FL‐HA, chitosan, MCTs. Again, although many were safe and effective for retinal tamponade, between both inflammatory reactions and the rapid clearance of few of these polymers, their effectiveness as a long‐term vitreous substitute is limited.

The use of liquids as a vitreous substitute may be more limited in terms of longer‐term toxicity. Although some positive outcomes were seen initially with PFO, PFP, F6H8, and O_62_, some patients ended up with recurrent RDs requiring reoperation. In terms of devices, FCVB is an emerging vitreous substitute for use in both complex and uncomplicated RD. Its implantation was able to successfully tamponade the retina, providing longer‐term retinal support with an ability to maintain IOP, offering a safe potential for the future of vitreous substitutes.

Beyond conventional ways to replace the vitreous, other possibilities under investigation include the artificial generation of vitreous in vitro, which could potentially address problems with artificial substitutes, including suitable biomechanical properties, biocompatibility, and challenges with nutrient transport. The complexity of the vitreal three‐dimensional structure presents a challenge to this effort. Early data from Sommer et al. shows potential by demonstrating that ascorbic acid enhances hyalocyte proliferation in a dose‐dependent manner at concentrations between 0.1–3 mg/mL by increasing collagen production and mRNA expression of cells in vitro, which has the potential to stimulate vitreous synthesis. However, indefinite hyalocyte proliferation is also undesirable and the control of hyalocyte growth can be regulated by specific growth factors (i.e., increased with bFGF and reduced by TGF‐B1) [53].

The other avenue for exploration has been vitreous transplantation. Shafer et al. transplanted vitreal tissue in a case series of 200 human vitreous transplants performed for RD. [54] Human vitreous was obtained via a stored eye‐bank aspirate stored at 4°C for 2–14 months, plated on blood agar and incubated for 48 h at 37°C prior to use. The vitreous was planted using an 18G needle through a pars plana incision with the needle tip just posterior to the recipient lens. Patients received daily antibiotic therapy with tetracycline for 4 days postoperatively. The retinal reattachment rate was 40% overall. The most frequent postoperative complication was mild vitreous haze that disappeared without treatment after 2–5 days. Uveitis occurred in 3.5% of cases and cleared after 2–9 months of corticosteroid therapy. In the transplants that failed to reattach the retina, regressive phenomena of cataracts, glaucoma, and phthisis occurred with the same frequency that would have been expected using other techniques. Shafer noted that the vitreous transplants lasted longer than air, but not saline. The implanted tissue showed a degradation time on the host, with a low inflammatory reaction. In addition, vitreous capsular devices in our review have shown some promise. These devices allow the injection of various conventional vitreous substitutes into the eye and prevent the interaction of the vitreous substitute with the bioenvironment of the ocular cavity, thereby aiming to reduce the toxic effects of the vitreous substitute. However, these devices have higher rates of complication, some without long‐term tamponade success and comparable biocompatibility with other substitutes.

Limitations of the present review include the stringent eligibility criteria for inclusion of studies. There was variability in the reporting of surgical techniques, preparation of animal models, and clinical, optical, and anatomical outcomes across included studies. Given that some included studies were published over 20 years ago, the routine practices in vitreoretinal surgery may have changed. Additionally, publication and language biases are possible given the nature of the inclusion criteria.

## 5. Conclusion

In conclusion, this systematic review aimed to provide a review of the biologic, physical, and clinical properties of novel vitreous substitutes from January 1990 to October 2025. Of 11,045 studies reviewed, 37 studies of novel substitutes were included. Novel experimental vitreous substitutes such as UV‐CHA, Vitargus ABV‐1701, PanaceaGel SPG‐178, E10KDC18, or FCVB show the most promising biological, optical, and physical properties for additional investigation as vitreous substitutes. 16/37 substitutes had > 80% success in tamponade of RDs (UV‐CHA, TCHA hydrogel, WTG‐127, EPC, PEG hydrogel, thiol‐containing hydrogel, CoP hydrogel, PanaceaGel SPG‐178, Vitargus ABV‐1701, HA‐PEG oxime‐crosslinked hydrogel, foldable capsular vitreous body (FCVB), E10KDC18, chitosan solution, PFO, F6H8, and O_62_). Twelve out of thirty‐seven substitutes showed high‐tissue biocompatibility (Healaflow, UV‐CHA, EPC, thiol‐containing hydrogel, CoP hydrogel, PanaceaGel SPG‐178, Vitargus ABV‐1701, HA‐PEG oxime‐crosslinked hydrogel, FCVB, E10KDC18, FL‐HA, and MCTs). Fifteen out of thirty‐seven substitutes showed low ocular inflammation (Healaflow, UV‐CHA, WTG‐127, PEG hydrogel, thiol‐containing hydrogel, CoP hydrogel, PanaceaGel SPG‐178, PVA/chitosan hydrogel, Vitargus ABV‐1701, HA‐PEG oxime‐crosslinked hydrogel, FCVB, E10KDC18, FL‐HA, Chitosan solution, and MCTs). Thirteen out of thirty‐seven substitutes showed optical properties similar to native vitreous (UV‐CHA, TCHA hydrogel, HPCTS‐ADA, WTG‐127, EPC, PEG hydrogel, thiol‐containing hydrogel, CoP hydrogel, PanaceaGel SPG‐178, Vitargus ABV‐1701, HA‐PEG oxime‐crosslinked hydrogel, E10KDC18, and O_62_). Fourteen out of thirty‐seven substitutes were associated with no significant changes in IOP (UV‐CHA, TCHA hydrogel, HPCTS‐ADA, WTG‐127, EPC, CoP hydrogel, PanaceaGel SPG‐178, HA‐PEG oxime‐crosslinked hydrogel, FCVB, E10KDC18, FL‐HA, chitosan solution, MCTs, and PFP). Further research is needed to investigate these substitutes as challenges with biocompatibility, tamponade success, and complications such as inflammation and elevated IOP pose challenges.

## Author Contributions

P.Y. conceived and supervised the study. D.S. and L.H. contributed to the study methodology, data extraction and interpretation, provided biomaterials expertise, and drafted and revised the manuscript. D.S. and L.H. contributed equally to this work. T.S.J., B.H., and E.L.Y. conducted the literature search, data extraction and interpretation, risk‐of‐bias assessments, and contributed to manuscript preparation. M.M.P., R.D., B.G.B., and P.Y. provided assessment of clinical relevance, expertise in vitreoretinal surgery and tamponades, and guidance on manuscript revisions. A.F. and M.S. contributed expertise in biomaterials and polymer science and assisted with the interpretation of vitreous substitute properties.

## Funding

No funding was received for this manuscript.

## Conflicts of Interest

The authors declare no conflicts of interest.

## Data Availability Statement

The authors confirm that the data supporting the findings of this study are available within the article and tables.
